# Gastric microbiome of Indian patients with *Helicobacter pylori* infection, and their interaction networks

**DOI:** 10.1038/s41598-017-15510-6

**Published:** 2017-11-13

**Authors:** Anubhav Das, Verima Pereira, Shruti Saxena, Tarini Shankar Ghosh, D. Anbumani, Satyabrata Bag, Bhabatosh Das, G. Balakrish Nair, Philip Abraham, Sharmila S. Mande

**Affiliations:** 1 Bio-Sciences R&D Division, TCS Research, Tata Consultancy Serviced Ltd., 54-B Hadapsar Industrial Estate, Pune, 411013 Maharashtra India; 2 Division of Gastroenterology, P D Hinduja Hospital, Mumbai, 400016 India; 3 Molecular Genetics Laboratory, Centre for Human Microbial Ecology, Translational Health Science and Technology Institute, Faridabad, 121001 India

## Abstract

The gastric microbiome is suspected to have a role in the causation of diseases by *Helicobacter pylori*. Reports on their relative abundance vis-à-vis *H. pylori* are available from various ethnic and geographic groups, but little is known about their interaction patterns. Endoscopic mucosal biopsy samples from the gastric antrum and corpus of 39 patients with suspected *H. pylori* infection were collected and microbiomes were analyzed by 16S rDNA profiling. Four groups of samples were identified, which harbored *Helicobacter* as well as a diverse group of bacteria including *Lactobacillus*, *Halomonas* and *Prevotella*. There was a negative association between the microbiome diversity and *Helicobacter* abundance. Network analyses showed that *Helicobacter* had negative interactions with members of the gastric microbiome, while other microbes interacted positively with each other, showing a higher tendency towards intra-cluster co-occurrence/co-operation. Cross-geographic comparisons suggested the presence of region-specific microbial abundance profiles. We report the microbial diversity, abundance variation and interaction patterns of the gastric microbiota of Indian patients with *H. pylori* infection and present a comparison of the same with the gastric microbial ecology in samples from different geographic regions. Such microbial abundance profiles and microbial interactions can help in understanding the pathophysiology of gastric ailments and can thus help in development of new strategies to curb it.

## Introduction

The acidic pH in the stomach lumen impedes bacterial growth^1^. However, it is now known that the human stomach is not sterile but is rather colonized by diverse microbiota^2^. High-throughput sequencing of gastric biopsy samples suggests that the human stomach may harbor 128 phyla, although mainly dominated by five phyla, namely, Proteobacteria, Firmicutes, Actinobacteria, Bacteroidetes and Fusobacteria^2–5^. Among these, several species of *Helicobacter* are known to be natural inhabitants of the human stomach. *Helicobacter pylori (H. pylori)*, a Gram-negative bacterium and frequent isolate of stomach specimens, with reported links to the causation of chronic/atrophic gastritis, duodenal ulcers, gastric mucosa-associated lymphoid tissue (MALT) lymphoma and adenocarcinoma^2,6,7^, has coevolved with its human host.

While *H. pylori* has been incriminated in disease, an aspect that has only been recently investigated is the role of the stomach microbiome in the causation of these diseases, especially adenocarcinoma. Studies focusing on the microbial composition of the stomach in healthy individuals^4,8–10^ have indicated the presence of genera like *Streptococcus*, *Prevotella*, *Veillonella*, *Fusobacterium*, *Haemophilus* and *Clostridium*. *H. pylori* is known to utilize specific molecular mechanisms to modulate host immune response and create a local micro-environment that aids its colonization^5,11^. The influence of *Helicobacter* abundance on the populations of other genera and *vice versa*, and any interactions between them in the causation of disease, is still being explored^5^.

In order to investigate this aspect, we analyzed stomach microbiota composition of 39 Indian patients with *H. pylori* infection, by investigating their 16S rRNA gene sequences. We compared the results with available sequences of the gastric microbiome from different geographic regions. This not only provided a comprehensive picture of the gastric microbiome in these individuals, but also highlighted the association of these groups with *Helicobacter* abundance.

## Results

### Subject demographics

The 39 subjects (aged 21 to 85 years; 23 men) included in the study were rapid urease test (RUT)-positive for *H. pylori*. None of the subjects was on proton pump inhibitors or antibiotics in the previous four weeks (Supplementary Table S1).

### Gastric microbial profiles

To investigate inter-individual variations in microbial community composition, microbial abundances for each sample were calculated based on the taxonomic assignments of the constituent V1-V5 rRNA gene sequences, followed by normalization across genera. Operational taxonomic unit (OTU)-level profiles of samples were also prepared by clustering the sequences into groups having >97% identity. Microbial entities present in at least 30% of the samples indicated a total of 50 genera and 70 OTUs. The lineages of each of the 70 OTUs have been listed in Supplementary Table S2. Also, the list of lowest level of classification corresponding to the taxa identified at the genera level is provided in Supplementary Table S3.

**Figure 1:**
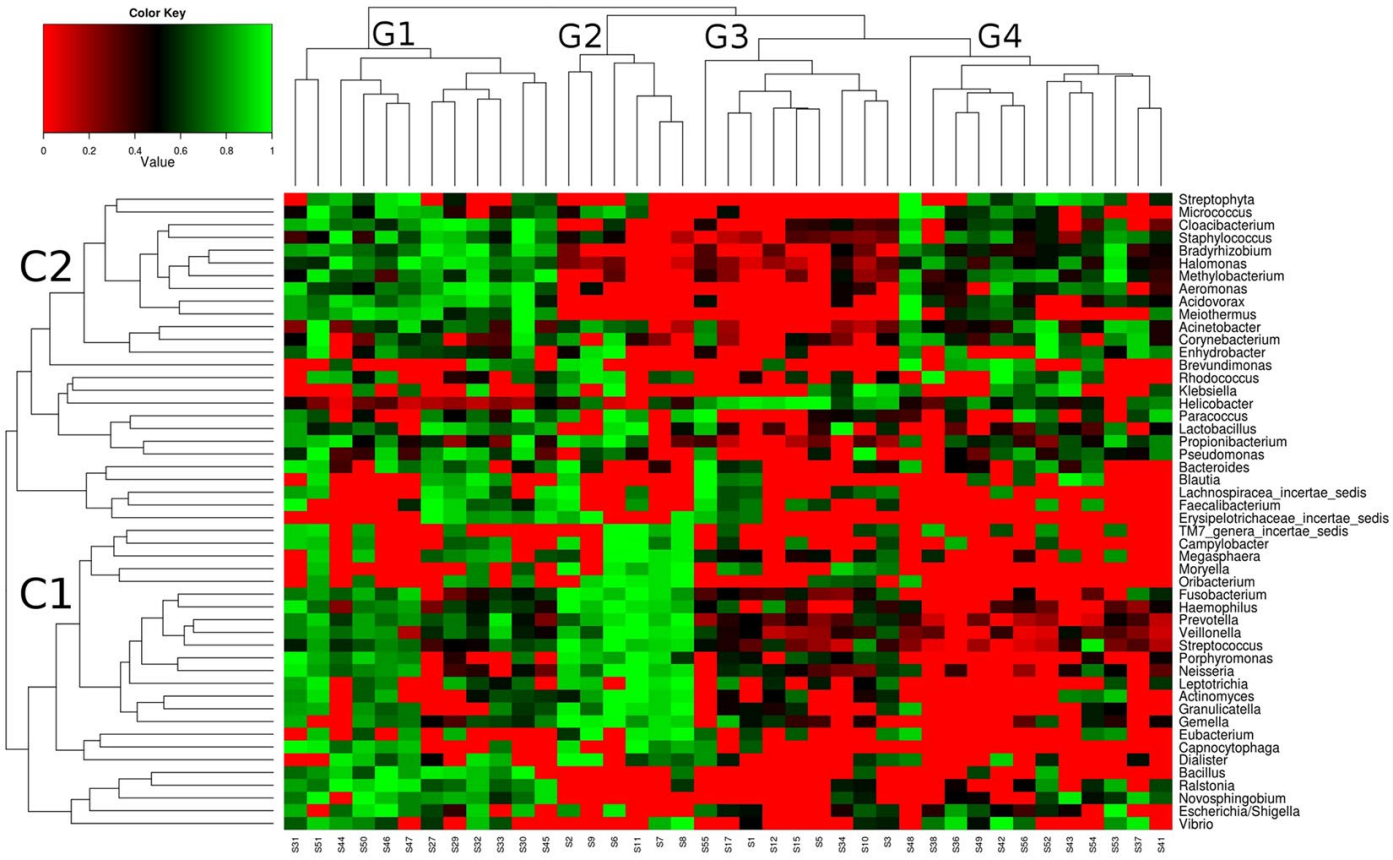
Microbial abundance profiles of 39 Indian subjects, showing inter-individual variations of microbial community structure. Genera abundances were calculated and normalized for each sample, followed by rank normalization across samples for each genus. Bi-clustering shows profile-based clustering of samples into 4 groups (G1 to G4) as well as microbial clades (Clusters C1 and C2).

Genera-level profiles were analyzed by bi-clustering using ranked abundances across samples that showed differential microbial abundance patterns (Fig. 1). Based on similarities in microbial abundance profiles, the samples could be clustered into four major groups (G1 to G4; Supplementary Table S1). Similarly, based on the abundances, the microbial genera in the samples could be classified into two main clusters (C1 and C2; Supplementary Table S4).

**Figure 2:**
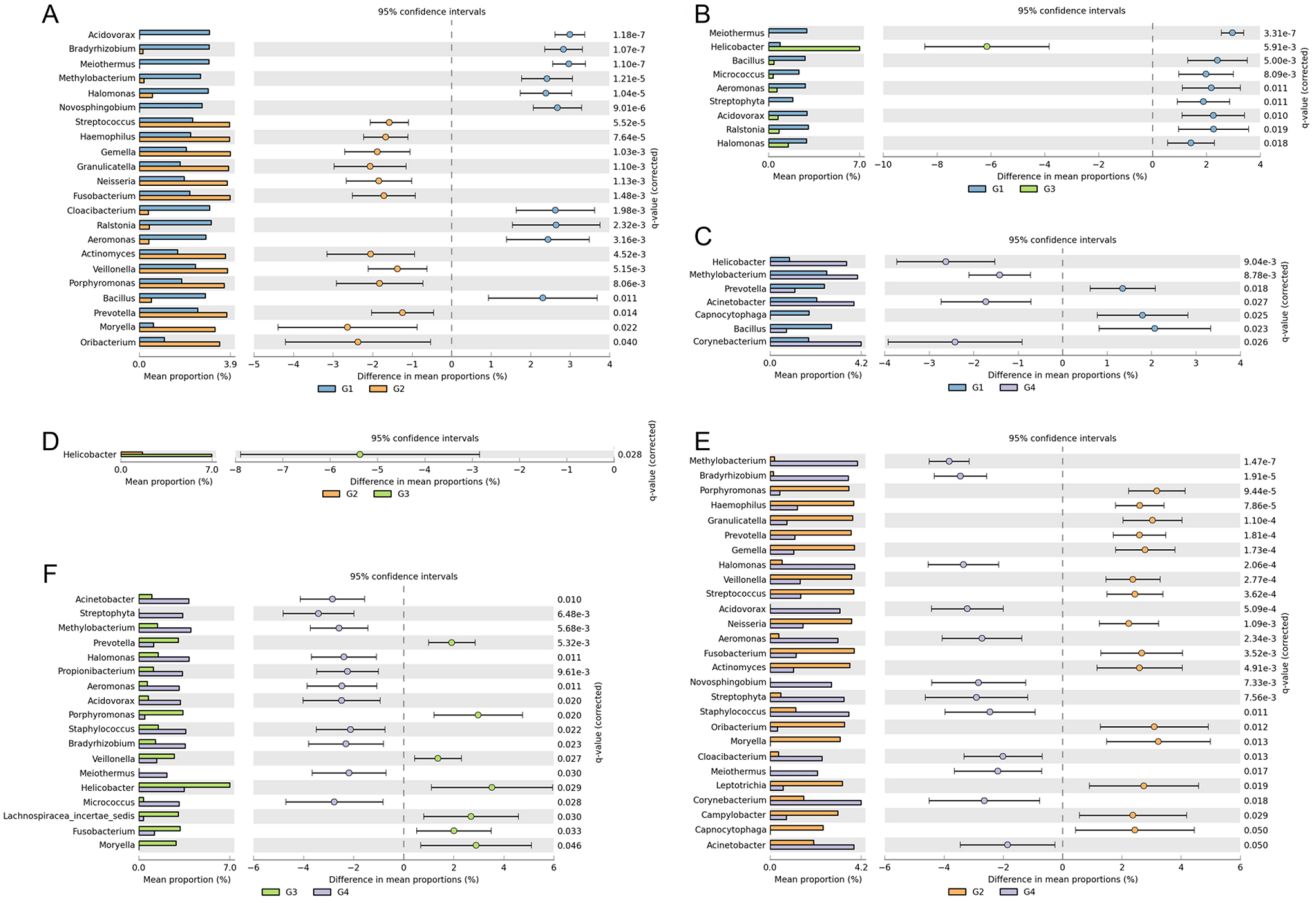
Pair-wise comparisons of microbial population of sample groups. (**A**) Comparison of microbial abundances between sample groups G1 and G2. (**B**) Comparison of microbial abundances between sample groups G1 and G3. (**C**) Comparison of microbial abundances between sample groups G1 and G4. (**D**) Comparison of microbial abundances between sample groups G2 and G3. (**E**) Comparison of microbial abundances between sample groups G2 and G4. (**F**) Comparison of microbial abundances between sample groups G3 and G4.

*Helicobacter* abundance pattern showed a distinct signature across the samples. Its abundance was highest in sample group G3, with intermediate presence in group G4. Sample groups G1 and G2 had least abundance of *Helicobacter*. A similar trend was reflected by OTU analyses (Supplementary Fig. S1), wherein, *Helicobacter* was primarily found to be abundant in sample group G3, followed by group G4. Pair-wise comparison between sample groups also showed distinguishing abundance patterns (Fig. 2).

### Helicobacter abundance and gastric microbial diversity

**Figure 3:**
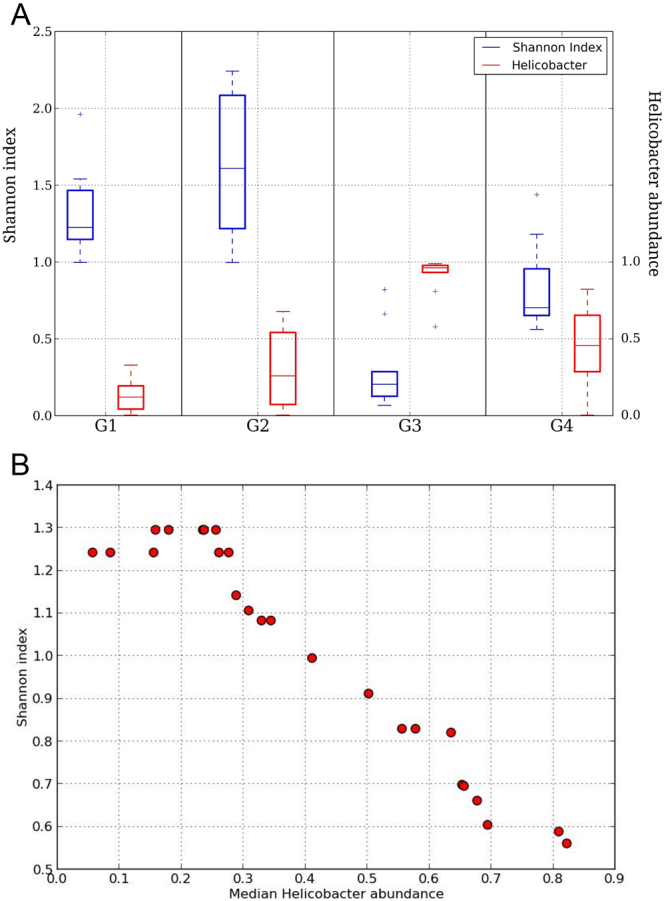
Variation of Shannon diversity with *Helicobacter* abundance. (**A**) Box plots showing variation in Shannon diversity with *Helicobacter* abundance in each sample group. (**B**) Scatter plot showing change in Shannon diversity with *Helicobacter* abundance.

Shannon diversity index of non-*Helicobacter* genera was calculated for each sample and variation was checked between sample groups (Fig. 3A). The highest median Shannon diversity was observed in groups G1 and G2 (low *Helicobacter* abundance). Sample groups having higher *Helicobacter* abundance (G3 and G4) had lower median Shannon diversity. Sample group G3 had the highest *Helicobacter* abundance with least Shannon diversity.

To investigate further, we adopted the sliding window approach^12^. For each sample, *Helicobacter* abundance and the corresponding Shannon diversity index were calculated. Samples were sorted in increasing order of *Helicobacter* abundance. Median *Helicobacter* abundance as well as corresponding median value of Shannon diversity for the first 15 samples was calculated. Keeping the window of 15 samples constant, the window was slid towards the higher values of *Helicobacter*. The resulting values were plotted as a scatter plot (Fig. 3B). While the Shannon diversity remained almost constant up to certain abundance of *Helicobacter* (~0.25), it showed gradual decrease with increase in *Helicobacter* abundance.

### Microbial interaction network

**Figure 4:**
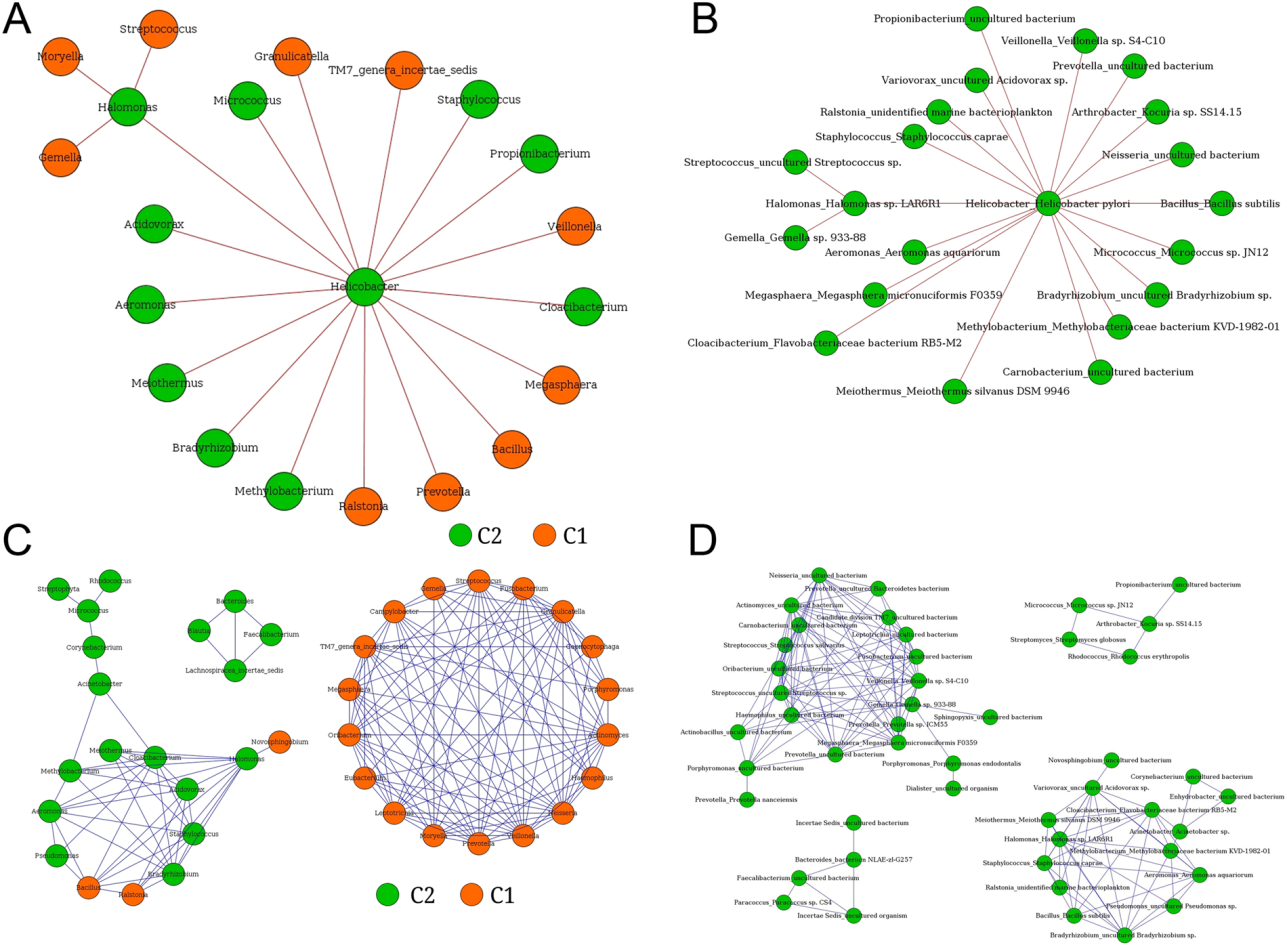
Interaction networks of microbes in the Indian samples. (**A**) Negative interaction network of microbial genera showing *Helicobacter* as lone hub. (**B**) Negative interaction network at OTU level showing similar trends and *H. pylori* acting as hub node. (**C**) Co-occurrence network showing interaction specificity of bacterial genera. Orange nodes correspond to microbes belonging to clade C1 and green nodes correspond to members of cluster C2. (**D**) Co-occurrence network of OTUs, showing similar associations.

The co-occurrence (indicative of positive interaction) and mutual exclusion (negative interaction) patterns among the microbial groups (at the level of genus and species) were investigated. Microbial correlation networks were generated using ReBoot method (see Methods). Analysis of significant negative interaction patterns revealed *Helicobacter* to be a core node with multiple negative interactions with various genera. Some major microbes that showed negative interactions with *Helicobacter* (Fig. 4A) included *Ralstonia* (belonging to cluster C1), *Bradyrhizobium* (C2), *Cloacibacterium* (C2), *Acidovorax* (C2), *Aeromonas* (C2), *Halomonas* (C2), *Bacillus* (C1), *Methylobacterium* (C2) and *Meiothermus* (C2). Similar trend was observed at the OTU level, where species corresponding to interacting genera also appeared to have negative interaction with *Helicobacter*, which acted as the hub of the network (Fig. 4B). The co-occurrence patterns revealed two major and separate networks, each dominated by members belonging to either cluster C1 or C2 (Fig. 4C). At the OTU level, the interaction patterns remained similar (Fig. 4D).

**Figure 5:**
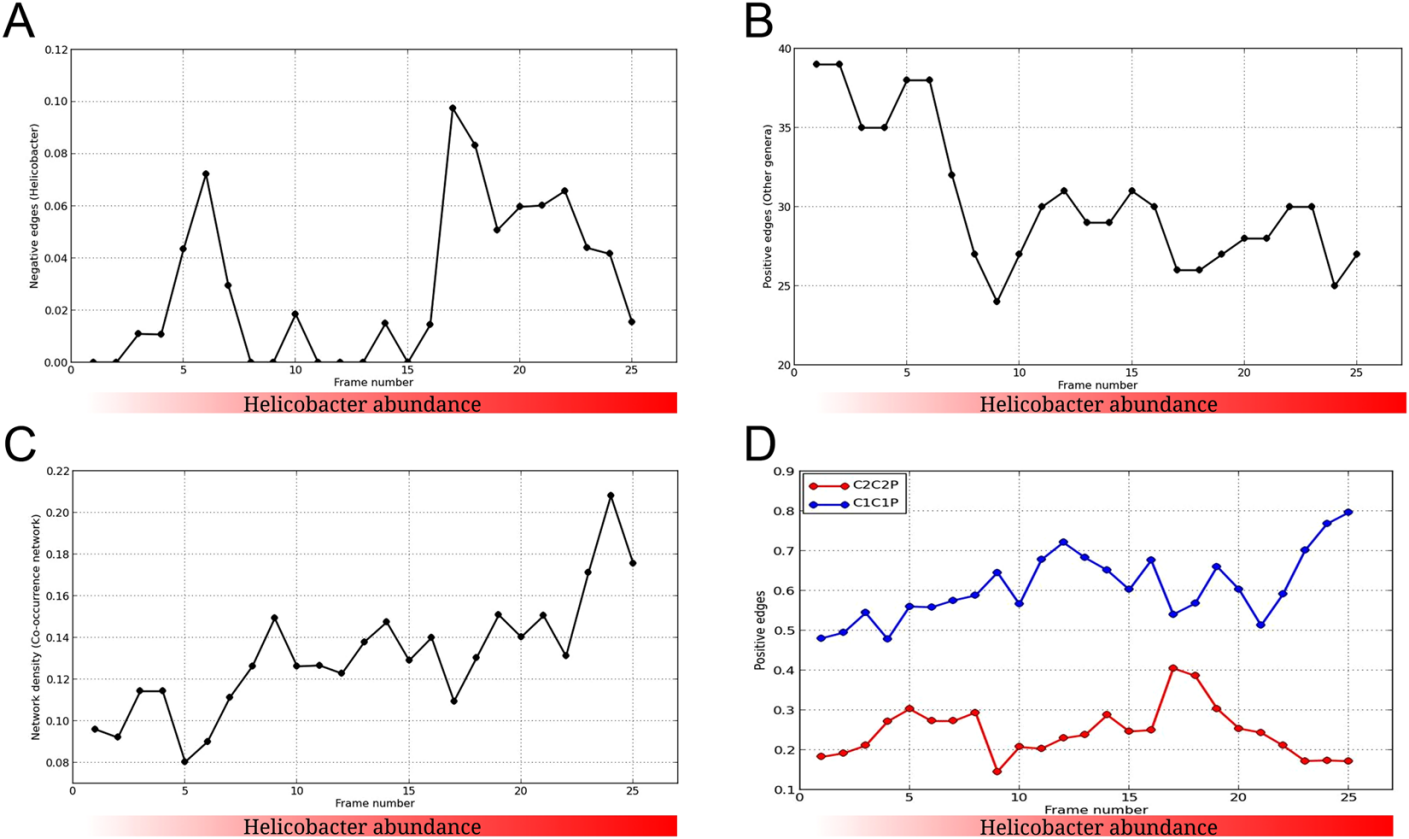
Variation of network properties with increase in *Helicobacter* abundance. (**A**) Variation of extent of negative interaction of *Helicobacter* with other microbes, with increase in its abundance. (**B**) Variation of cooperation between microbes other than *Helicobacter* with increase in *Helicobacter* abundance. (**C**) Change in density of microbial network of genera other than *Helicobacter* with increase in *Helicobacter* infection. (**D**) Clade-specific co-operation variation with increase in *Helicobacter* abundance.

Apart from overall interaction patterns, the effect of change in *Helicobacter* abundance on microbial interactions was evaluated by performing network analysis using the sliding window approach^12^. Based on the 25 frames of 15 samples each, the networks and their properties were checked. Two peaks representing noticeably higher number of negative interactions between *Helicobacter* and other microbial genera were observed. While one peak corresponded to negative interactions in samples corresponding to lower abundance of *Helicobacter*, the second peak indicated similar negative interactions in samples with higher *Helicobacter* abundance (Fig. 5A), suggesting a possible two-way modulation of bacterial abundance. While the total number of contributing genera, other than *Helicobacter*, showed a decrease with *Helicobacter* abundance, the network density of the same was observed to increase (Fig. 5B,C).

The variation of intra-cluster positive interactions of bacterial genera also showed distinct behavior (Fig. 5D). For this, the number of positive interactions between genera belonging to clusters C1 and C2 were calculated separately for each frame and plotted against the same. Members of C1 had more interactions than those of C2. The number of positive interactions between members of C1 appeared to increase at lower levels of *Helicobacter*, followed by saturation at intermediate *Helicobacter* abundance, and finally increase sharply at higher abundance of *Helicobacter*. While the sensitivity of cluster C1 was observed to be less, members of cluster C2 showed increased responsiveness to *Helicobacter*. The variation of positive interactions between members of C2 showed trend similar to that of negative interaction patterns of *Helicobacter* with its abundance (Fig. 5A). These results suggest that the interactions/associations between various microbial genera are modulated by the presence of *Helicobacter*.

### Variation of stomach microbial profile with geography

**Figure 6:**
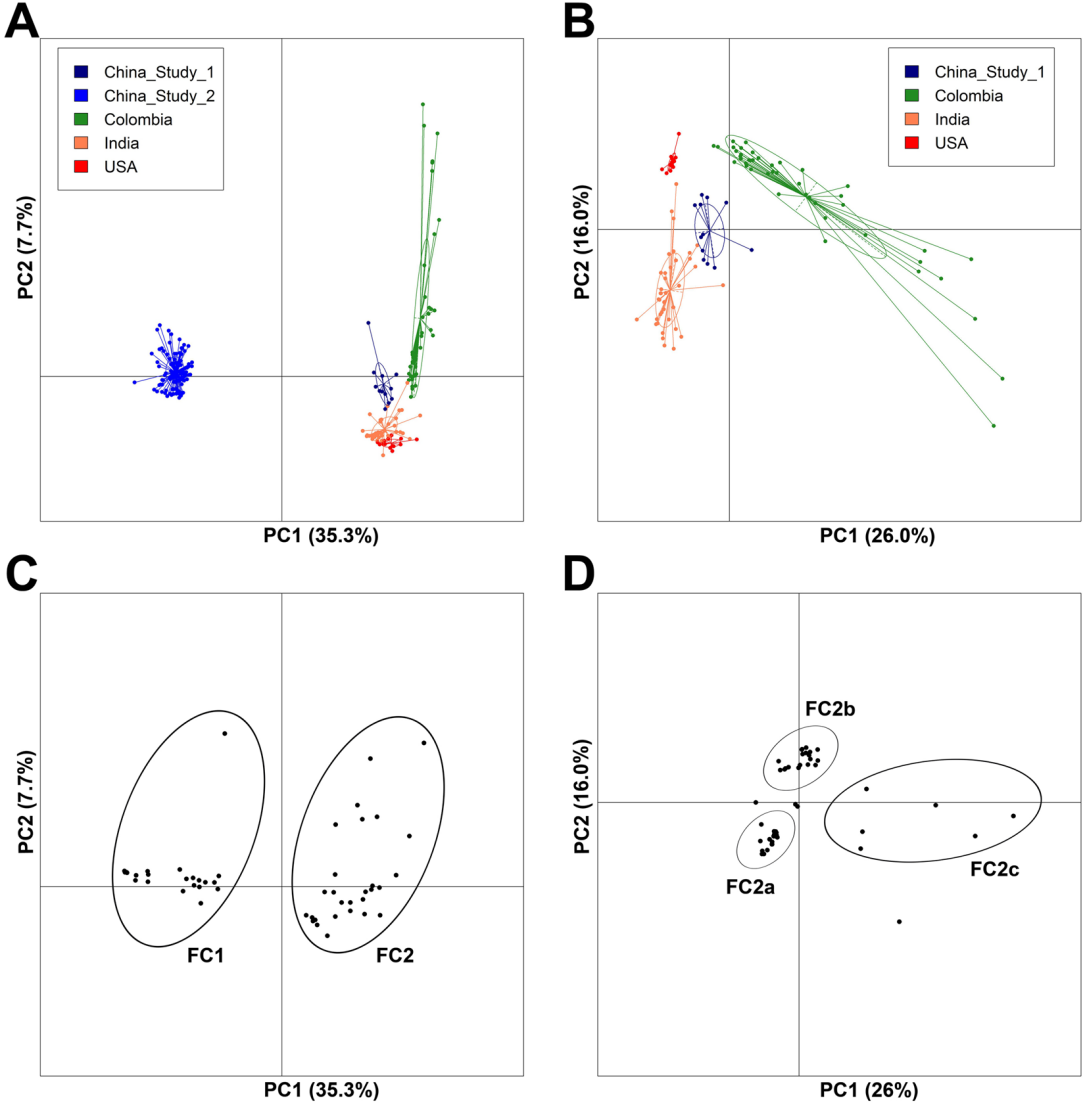
Cross-geography comparison of microbial profiles using Principle Component Analyses (PCA). (**A**) PCA of publicly available samples from four studies and the reported Indian cohort showing broad separation of a Chinese dataset comprising samples from patients with advanced gastric cancer. (**B**) PCA of publicly available samples from three studies (gastric cancer patient samples ignored) and the Indian cohort, showing geography-based clustering. (**C**) Loadings of features in PCA of publicly available samples from four studies and the reported Indian cohort showing broad separation of one Chinese dataset comprising samples from patients with advanced gastric cancer. (**D**) Loadings of features in PCA of publicly available samples from three studies (gastric cancer patient samples ignored) and the Indian cohort, showing geography-based clustering.

In order to evaluate whether the gastric microbial community varies between individuals from different geographic locations, similar to that observed with the gut microbiome, available gastric microbiome datasets were downloaded from NCBI and analyzed. These were obtained from bio-projects bearing IDs corresponding to the country of origin, namely, PRJNA270661 (USA)^13^, PRJNA289186 (China_Study_1), PRJNA313391 (China_Study_2) and PRJEB11763 (Colombia)^14^. Principle component analyses (PCA) of the data from Indian samples and these datasets showed a strong separation of one set of Chinese data (Chinese_Study_2) (Fig. 6A), corresponding to samples collected from subjects suffering from advanced gastric cancer. A possible reason for such a distinct separation could be due to the usage of different sequencing platforms. While Chinese_Study_2 samples were sequenced on Illumina, other studies including the Indian samples were sequenced on 454 FLX platform. PCA of samples excluding the Chinese_Study_2 dataset showed distinct region-specific clustering patterns of the samples (Fig. 6B). Based on PCA loadings of features using all samples, 2 distinct feature clusters, viz., FC1 and FC2 were identified (Fig. 6C and Supplementary Fig. S2). While FC1 comprised of bacterial genera exclusive to Chinese_Study_2, FC2 members appeared to be drivers in rest of the samples. PCA loadings of features using samples other than Chinese_Study_2 showed further clustering of FC2 members, viz., FC2a, FC2b, and FC2c (Fig. 6D and Supplementary Fig. S3). Based on the ranked median abundances of various genera in samples from each region, samples from USA and Colombia were found to cluster together and those from the Indian samples (current study) and the Chinese_Study_1 samples appeared closer to each other (Supplementary Fig. S4).

Microbial interaction network analyses of the additional publicly available datasets indicated presence of members of only cluster C1 (and not C2) as major players in other region-specific genera. This suggests that the C1 members form a common core microbiome that are present in subjects from various geographic regions and may be critical in determining the regional basis of variation in microbial community dynamics (Supplementary Figs S5–S8). Similar and dense co-occurrence patterns of members of C1 in Chinese_Study_1 and the Colombian as well as Indian studies (Fig. 4C, Supplementary Figs S6, S8) suggest that members of C1 tend to co-operate substantially with each other and are probably related to *Helicobacter* abundance.

Dominance of *Helicobacter* was observed in mutual exclusion networks of all the studies containing *Helicobacter* in the corresponding samples (Supplementary Figs S9–S12). *Helicobacter* appeared to be the hub in these study-specific networks, similar to that observed in the present study with Indian patients. Interestingly, in Chinese_Study_2 (Supplementary Fig. S11), where *Helicobacter* was absent and had a mutually exclusive set of microbes, although negative interactions (similar to other studies) were seen, it was dominated by *Wolinella*, which acted as the hub in the network. *Wolinella* belongs to the same family as *Helicobacter*, i.e., Helicobacteraceae. This suggests that other members of the Helicobacteraceae family can function similar to *Helicobacter*. The change in Shannon diversity with change in *Wolinella* and *Helicobacter* abundance (Supplementary Figs S13, S14), as observed in Chinese_Study_2 and the Colombian study, showed variations similar to that in the Indian samples. This further suggests similar behavior of *Helicobacter* (irrespective of region and samples), and also strengthens the observation of *Wolinella* being capable of functioning like *Helicobacter*.

## Discussion

The present study reports, for the first time, a comprehensive investigation of the microbial community in the stomach of Indian patients with *H. pylori* infection. The existence of a diverse microbiome in the stomach of subjects, as has also been documented in other recent studies^2^, suggests that the human stomach is host to a far richer microbial population than was earlier believed. Faust *et al*.^15^ reviewed the mechanisms by which organisms in a body niche may interact to each other’s benefit or detriment in the context of disease production. We undertook this study to determine the interaction between coexisting organisms in the stomach harboring *H. pylori*.

Studies have looked into perturbances in the gastric microbiome in individuals harboring *H. pylori* in the presence or absence of disease (gastritis, peptic ulcer disease, and gastric cancer), in humans as well as in animals^16^. While species differ, it seems quite clear that *H. pylori* tends to predominate (when present), but its abundance may influence and be influenced by coexisting gastric microbiome. It is reasonable to assume that while disease manifestation may largely be the result of the pathogenicity of one species (in this case, *H. pylori*), the final outcome may depend also on the network of coexisting microbiota.

The present study indicates an inverse relationship between microbial diversity and *Helicobacter* abundance as well as probable modulations of microbial abundance by *Helicobacter*, similar to those reported previously^2,5^. Based on abundances, we could divide our samples into two main clusters and four major groups. The highest Shannon diversity was observed in the groups with low *Helicobacter* abundance; contrarily, groups with higher *Helicobacter* abundance had lower Shannon diversity. While the Shannon diversity remained almost constant up to certain abundance of *Helicobacter* (~0.25), it showed gradual decrease with increase in *Helicobacter* abundance, suggesting a link between microbial diversity and *Helicobacter* abundance.

The close interactions of *Helicobacter* with other genera as well as co-occurrence patterns of members of the cluster C2 (Supplementary Table S4) with *Helicobacter* dominance indicate that members of the cluster C2 are probably more critical than members of C1 (Supplementary Table S4) in driving the microbial community dynamics of the gastric microbiome.

Several possible mechanisms have been proposed for co-occurrence as well as mutual exclusion of organisms in body sites^15^. *H. pylori*, for example, secretes 20-amino-acid-long cecropin-like antibacterial peptides^17^. These peptides can also induce a pro-inflammatory response^18^, which may lead to local acidosis. As is well known, *H. pylori* stimulates gastrin release^19^; the resultant initial reduction in pH of the gastric environment makes the stomach inhospitable for microbes. On the other hand, rise in the pH of the gastric environment that can follow chronic infection by *H.pylori* (resulting in the development of atrophic gastritis) may provide optimal growth conditions to microbial genera that are generally incapable of growing in acidic environments. This could lead to a microbial bloom that can outnumber *Helicobacter*. The increased microbial growth may further inhibit the growth of *Helicobacter* through nutritional competition or by yet unknown mechanisms. This may explain the observed inverse correlation between *Helicobacter* abundance and the diversity of the coexistent microbes.

Analysis of microbial interaction network showed *Helicobacter* to be the core node having multiple negative interactions with various genera, most of which belong to cluster C2. The negative interactions occurred in two peaks, one with lower *Helicobacter* abundance and the other with higher abundance. Cluster C2 showed an increased responsiveness to *Helicobacter*. Intra-cluster interactions were observed to be more in cluster C1, especially at lower levels of *Helicobacter* abundance.

It must, however, be remembered that not all observed positive (co-occurrence) or negative (mutual-exclusion) relationships between organisms may occur in actual scenario, since these predictions are based on correlations, which may be statistically robust but biologically not feasible. Additional validations using experimental techniques are expected to provide robust set of microbial interactions.

Microbial interaction network analyses using publicly available datasets suggest that members of cluster C1 form common interaction core in datasets from various geographic regions and may be critical in determining the regional basis of variation in microbial community dynamics. Members of C1 tend to co-operate substantially with each other and are probably related to *Helicobacter* abundance. In a Chinese dataset (samples from patients with gastric cancer), although *Helicobacter* was found to be absent, *Wolinella* (another member of the Helicobacteraceae family) was seen to be dominated and acted as the hub of the network, suggesting its capability to function like *Helicobacter*.

Although therapeutic approach to *H. pylori* infection currently focuses on antimicrobial strategies, means to modulate the stomach microbiota may be another approach worth considering, either for prevention or for treatment.

To summarize, our results present a comprehensive and novel perspective on the microbial communities in the stomach ecosystem. The analyses of cross-geography microbial composition and interaction further provided insights into the regional basis of microbiome variation. The observed interaction patterns (both positive and negative) can help in identifying microbial genera that may be used to restore harmony in the gastric microbial community, thereby leading to alternative strategies to curb the gastric dysbiosis caused by *Helicobacter* and other pathogenic genera.

## Methods

### Sample collection and DNA extraction

Patients who were undergoing gastroscopy for indications decided by their treating physician, and who consented to providing biopsy specimens for this study, were screened. The study protocol was approved by the institutional review board of P D Hinduja Hospital (Project No. 760-PA-13), where the endoscopies were done. All experiments were performed in accordance with relevant guidelines and regulations recommended by the institution and informed consents were obtained from all subjects and/or their legal guardians prior to sample collection. Biopsy samples were collected from the gastric antrum and corpus. Samples from 39 patients who tested positive for *H. pylori* infection by the bedside RUT were included for analysis. The samples were collected aseptically in 0.9% sterile saline and transported to the laboratory in cold condition within 30 minutes of collection.

Community microbial genomic DNA was extracted from each biopsy sample using previously reported extraction methods^20^. Briefly, a combination of enzymatic, chemical and mechanical lysis methods was employed to ensure maximum lysis of bacterial cells. Mutanolysin, lysozyme and lysostaphin, the glycosidases and endopeptidase specific for microbial cell wall, were used to remove the cell wall from both Gram-positive and Gram-negative bacteria. Mechanical lysis was done by bead beating using 0.1 mm zirconium beads and a mini bead-beater (Analytik Jena, Germany). The genomic DNA was precipitated at room temperature by organic extraction using 0.8 volume of 100% isopropanol. The quality and quantity of the isolated genomic DNA were measured by spectrophotometer and integrity was checked by agarose gel electrophoresis.

### 16S rRNA gene sequencing

Amplification of the V1-V5 region of the SSU ribosomal 16S rRNA gene was performed using forward and reverse primers specific for C1 (27 F) and C5 (926 R). The amplicon of each sample was labeled with 5–6 nucleotides barcode and sequencing primer-specific adapter sequence (Supplementary Table S5). Amplification reaction was carried out in 50 microliter reaction volume with 10 ng of template DNA extracted from the biopsy samples. The PCR amplicons were purified using QIA quick gel elution kit (Qiagen, Germany). Equimolar libraries were pooled and sequenced using a 454 GS FLX+ pyrosequencer platform at the Centre for Human Microbial Ecology at the Translational Health Science and Technology Institute. The sequencing runs yielded 941,415 reads with average read length of 848 nucleotides.

### Data processing and taxonomic profiling

Initial filtering of obtained sequences was performed using quality information. A moderate threshold of average quality >25 was applied to filter the sequences. Of the 941,415 reads, 940,591 reads were retained, achieving >99% recovery. The filtered reads were then split into samples using sample-specific tags and *NGSTagCleaner* software^21^. A detailed summary of the number of sequences in respective samples is provided in Supplementary Table S5.

Taxonomic assignments of reads were performed using RDP-classifier V2.8 with a cut-off of 0.8^22^. Abundance table at genus level was generated and normalized within samples for further analyses. In order to identify the species-level phylotypes, filtered sequences were pooled and clustered into operational taxonomic units (OTU) using CROP^23^, with an identity cut-off of 97%. The taxonomic assignments of the representative sequences of the clusters obtained were done by performing *BLASTn* search of these sequences with stringent filtering cut-offs (identity percentage >97%, query coverage of 100%) against the SILVA database^24^. The classification lineage of OTU representatives are listed in Supplementary Table S2.

### Analyses of interaction networks of constituent microbial taxa

Pair-wise correlation coefficients (negative indicating mutual exclusion and positive indicating co-occurrence) between the normalized abundances of different genera were first obtained. The genera pairs having statistically significant correlation coefficients (corrected p value after Benjamini Hochberg FDR test; p < 0.05) were identified using the ReBoot method^15^ and were converted into networks. The networks were subsequently visualized using the *y*EDgraph editor^25^.

### Statistical analyses

Statistical analyses were performed using STAMP^26^. Pair-wise comparisons were tested for significance using Welch’s *t* test corrected by the Benjamini-Hochberg FDR method. For multiple groups, significance was tested by performing two-sided Kruskal Wallis H test followed by the Benjamini-Hochberg FDR correction. Corrected p value < 0.05 was considered to be statistically significant.

### Data availability

The NGS datasets have been submitted at NCBI (https://www.ncbi.nlm.nih.gov/Traces/study/?acc=SRP117557).

## Electronic supplementary material

Supplementary information
