# Supplementary material for: Gastric microbiome of Indian patients with Helicobacter pylori infection, and their interaction networks
Source: Sci Rep. 2017 Nov 13;7:15438. doi: 10.1038/s41598-017-15510-6 (PMC5684312; doi:10.1038/s41598-017-15510-6)
Supplement: Supplementary file 1 — Supplementary information [file 41598_2017_15510_MOESM1_ESM.pdf]

Gastric microbiome of Indian patients with *Helicobacter pylori* infection, and their interaction networks

Anubhav Das[1#], Verima Pereira[2#], Shruti Saxena[3#], Tarini Shankar Ghosh[1], Anbumani D[3], Satyabrata Bag[3], Bhabatosh Das[3], G. Balakrish Nair[3], Philip Abraham[2*] , Sharmila S Mande[1*]

[1] Bio-Sciences R&D Division, TCS Research, Tata Research Development & Design Centre, Pune 411013, India

[2] Division of Gastroenterology, P D Hinduja Hospital, Mumbai 400016, India

[3] Molecular Genetics Laboratory, Centre for Human Microbial Ecology, Translational Health Science and Technology Institute, Faridabad 121001, India

# These authors have contributed equally

***Correspondence to:**

Dr. Sharmila S Mande, Bio-Sciences R&D Division, TCS Research, Tata Research Development & Design Centre, 54-B, Hadapsar Industrial Estate, Pune 411013, India. E-mail: sharmila@atc.tcs.com. Fax: +91 2066086399. Phone: +912066086432

Dr. Philip Abraham, Division of Gastroenterology, P D Hinduja Hospital, Mahim, Mumbai 400016, India. E-mail: dr_pabraham@hindujahospital.com. Fax: +91 22 24440425. Phone: +91 22 24447104

The following supplementary data includes information which has been used in this study. The supplementary figure and legends along with supplementary tables and their respective titles are as follows.

Supplementary information

Supplementary Figure S1: OTU level abundance profile of the reported Indian cohort.

Supplementary Figure S2: Heat map showing PCA loadings of genera onto the first 2 principal components, for all the samples.

Supplementary Figure S3: Heat map showing PCA loadings of genera onto the first 2 principal components, for samples other than Chinese_Study_2.

Supplementary Figure S4: Heatmap of ranked median abundances of each microbial genera present in the considered studies, showing geography-based clustering.

Supplementary Figure S5: Co-occurrence network of microbial genera present in samples from USA.

Supplementary Figure S6: Co-occurrence network of microbial genera present in samples from Chinese study 1.

Supplementary Figure S7: Co-occurrence network of microbial genera present in samples from Chinese study 2.

Supplementary Figure S8: Co-occurrence network of microbial genera present in samples from Colombian study.

Supplementary Figure S9: Negative interaction network of microbial genera present in samples from USA.

Supplementary Figure S10: Negative interaction network of microbial genera present in samples from Chinese study 1.

Supplementary Figure S11: Negative interaction network of microbial genera present in samples

from Chinese study 2.

Supplementary Figure S12: Negative interaction network of microbial genera present in samples from Colombian study.

Supplementary Figure S13: Variation of Shannon diversity with Wolinella abundance, as calculated for Chinese study 2.

Supplementary Figure S14: Variation of Shannon diversity with Helicobacter abundance, as calculated for Colombian study.

Supplementary Table S1: Sample IDs of subjects of reported Indian cohort, with corresponding age and gender information along with group affiliation.

Supplementary Table S2: Taxonomic lineage from classification of Operational Taxonomic Units (OTUs).

Supplementary Table S3: Microbial genera and corresponding lowest taxa affiliation identified at OTU level.

Supplementary Table S4: Microbial genera identified in reported Indian cohort and corresponding cluster affiliations.

Supplementary Table S5: Sample ids and sequencing information of each sample.

Supplementary Figure S1: OTU level abundance profile of the reported Indian cohort.

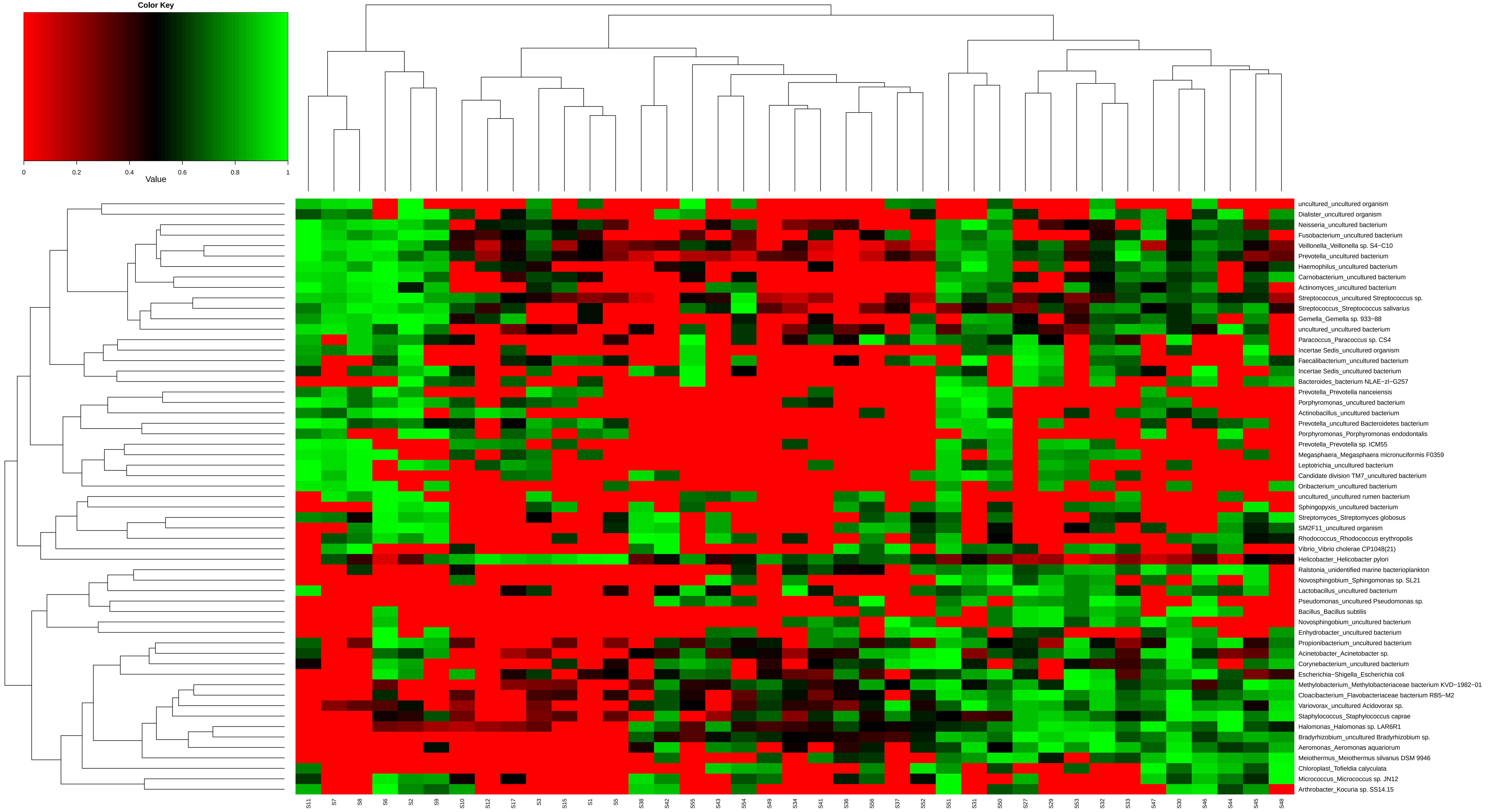

Color Key
0
0.2
0.4
0.6
0.8
1
Value
uncultured_uncultured organism
Dialister_uncultured organism
Neisseria_uncultured bacterium
Fusobacterium_uncultured bacterium
Veillonella_Veillonella sp. S4−C10
Prevotella_uncultured bacterium
Haemophilus_uncultured bacterium
Carnobacterium_uncultured bacterium
Actinomyces_uncultured bacterium
Streptococcus_uncultured Streptococcus sp.
Streptococcus_Streptococcus salivarius
Gemella_Gemella sp. 933−88
uncultured_uncultured bacterium
Paracoccus_Paracoccus sp. CS4
Incertae Sedis_uncultured organism
Faecalibacterium_uncultured bacterium
Incertae Sedis_uncultured bacterium
Bacteroides_bacterium NLAE−zl−G257
Prevotella_Prevotella nanceiensis
Porphyromonas_uncultured bacterium
Actinobacillus_uncultured bacterium
Prevotella_uncultured Bacteroidetes bacterium
Porphyromonas_Porphyromonas endodontalis
Prevotella_Prevotella sp. ICM55
Megasphaera_Megasphaera micronuciformis F0359
Leptotrichia_uncultured bacterium
Candidate division TM7_uncultured bacterium
Oribacterium_uncultured bacterium
uncultured_uncultured rumen bacterium
Sphingopyxis_uncultured bacterium
Streptomyces_Streptomyces globosus
SM2F11_uncultured organism
Rhodococcus_Rhodococcus erythropolis
Vibrio_Vibrio cholerae CP1048(21)
Helicobacter_Helicobacter pylori
Ralstonia_unidentified marine bacterioplankton
Novosphingobium_Sphingomonas sp. SL21
Lactobacillus_uncultured bacterium
Pseudomonas_uncultured Pseudomonas sp.
Bacillus_Bacillus subtilis
Novosphingobium_uncultured bacterium
Enhydrobacter_uncultured bacterium
Propionibacterium_uncultured bacterium
Acinetobacter_Acinetobacter sp.
Corynebacterium_uncultured bacterium
Escherichia−Shigella_Escherichia coli
Methylobacterium_Methylobacteriaceae bacterium KVD−1982−01
Cloacibacterium_Flavobacteriaceae bacterium RB5−M2
Variovorax_uncultured Acidovorax sp.
Staphylococcus_Staphylococcus caprae
Halomonas_Halomonas sp. LAR6R1
Bradyrhizobium_uncultured Bradyrhizobium sp.
Aeromonas_Aeromonas aquariorum
Meiothermus_Meiothermus silvanus DSM 9946
Chloroplast_Tofieldia calyculata
Micrococcus_Micrococcus sp. JN12
Arthrobacter_Kocuria sp. SS14.15
S11
S7
S8
S6
S2
S9
S10
S12
S17
S3
S15
S1
S5
S38
S42
S55
S43
S54
S49
S34
S41
S36
S56
S37
S52
S51
S31
S50
S27
S29
S53
S32
S33
S47
S30
S46
S44
S45
S48

Supplementary Figure S2: Heat map showing PCA loadings of genera onto the first 2 principal components, for all the samples.

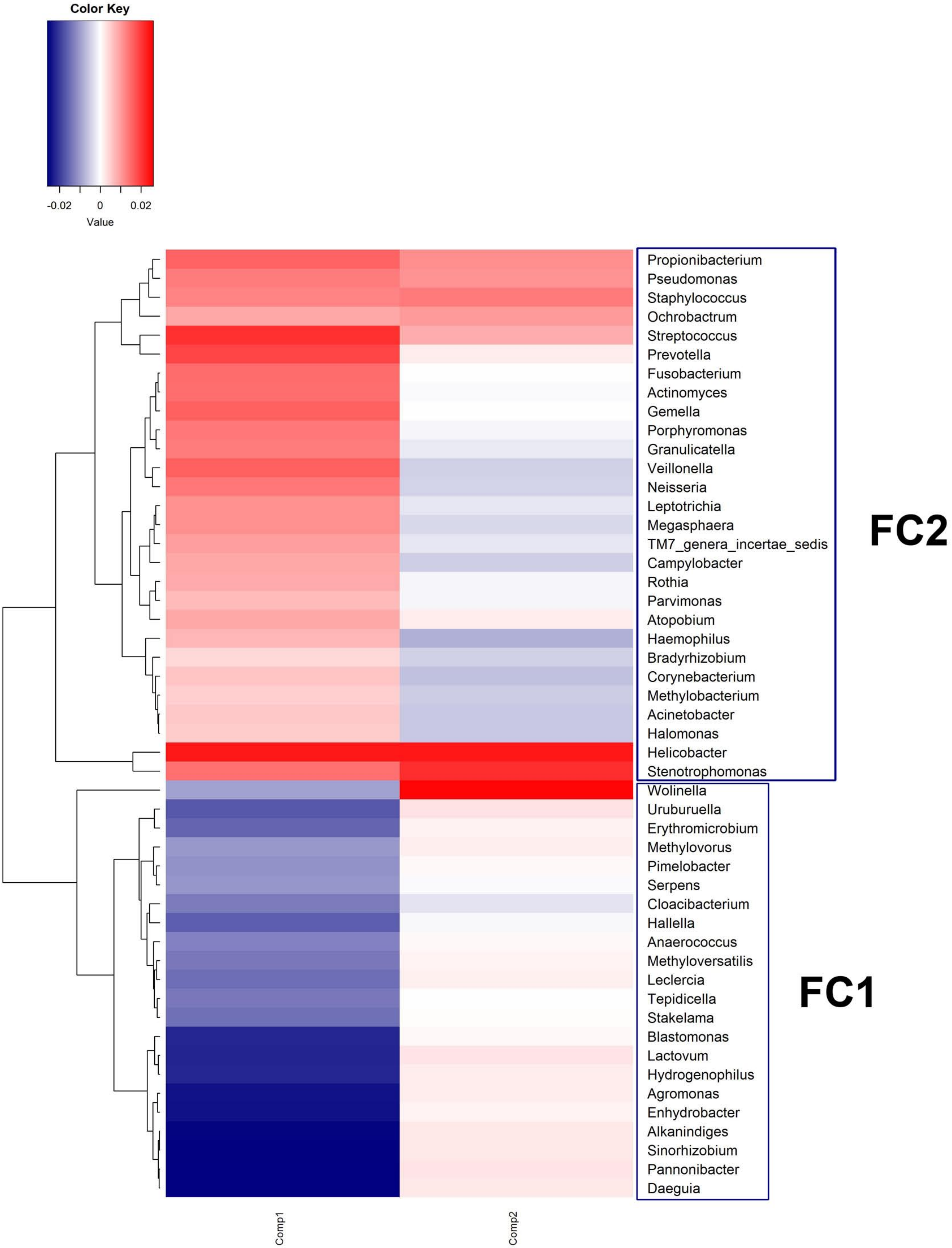
Color Key
-0.02
0
0.02
Value
Propionibacterium
Pseudomonas
Staphylococcus
Ochrobactrum
Streptococcus
Prevotella
Fusobacterium
Actinomyces
Gemella
Porphyromonas
Granulicatella
Veillonella
Neisseria
Leptotrichia
Megasphaera
TM7_genera_incertae_sedis
Campylobacter
Rothia
Parvimonas
Atopobium
Haemophilus
Bradyrhizobium
Corynebacterium
Methylobacterium
Acinetobacter
Halomonas
Helicobacter
Stenotrophomonas
Wolinella
Uruburuella
Erythromicrobium
Methylovorus
Pimelobacter
Serpens
Cloacibacterium
Hallella
Anaerococcus
Methyloversatilis
Leclercia
Tepidicella
Stakelama
Blastomonas
Lactovum
Hydrogenophilus
Agromonas
Enhydrobacter
Alkanindiges
Sinorhizobium
Pannonibacter
Daeguia
FC2
FC1
Comp1
Comp2

Supplementary Figure S3: Heat map showing PCA loadings of genera onto the first 2 principal components, for samples other than Chinese_Study_2.

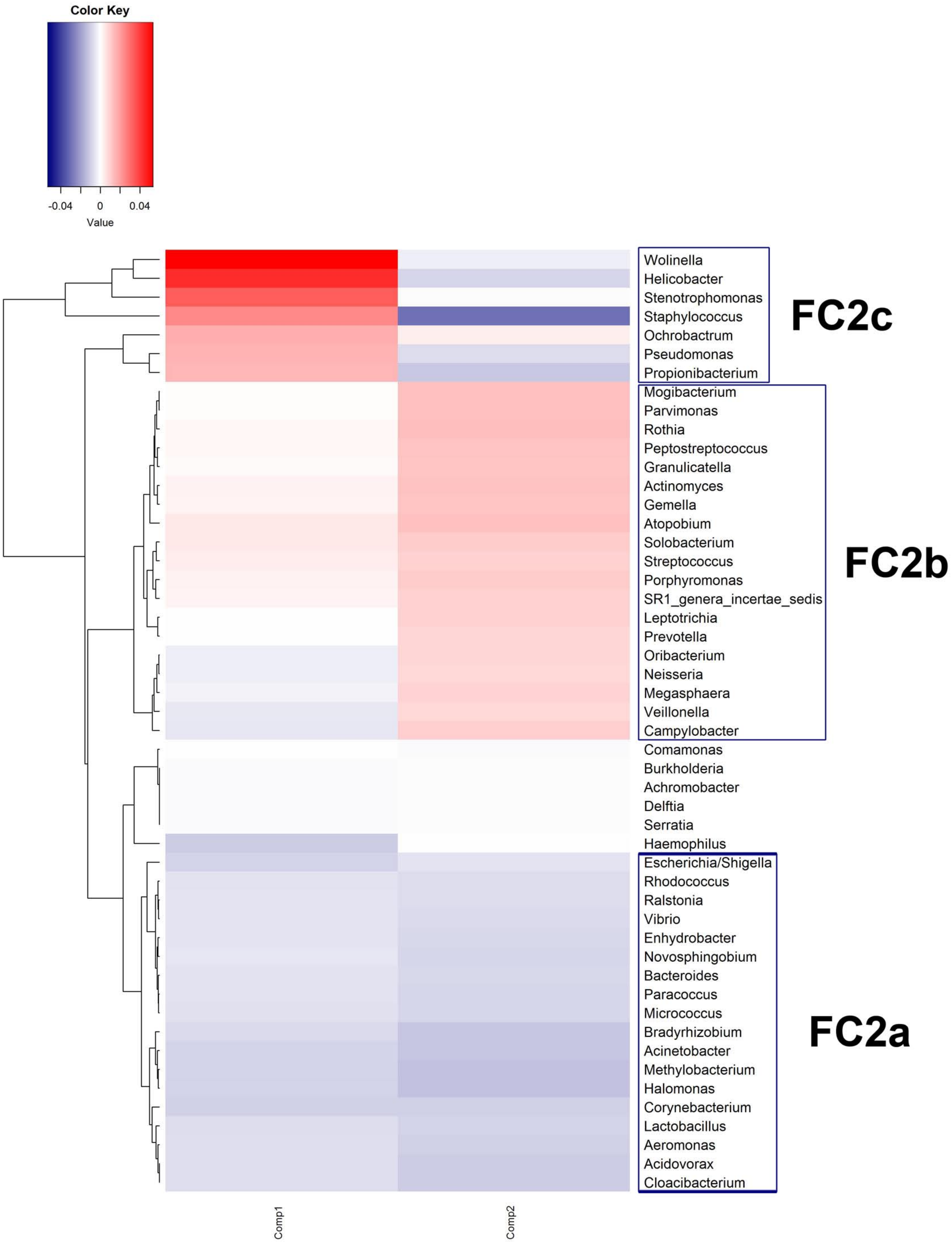
Color Key
-0.04
0
0.04
Value
Wolinella
Helicobacter
Stenotrophomonas
Staphylococcus
Ochrobactrum
Pseudomonas
Propionibacterium
FC2c
Mogibacterium
Parvimonas
Rothia
Peptostreptococcus
Granulicatella
Actinomyces
Gemella
Atopobium
Solobacterium
Streptococcus
Porphyromonas
SR1_genera_incertae_sedis
Leptotrichia
Prevotella
Oribacterium
Neisseria
Megasphaera
Veillonella
Campylobacter
FC2b
Comamonas
Burkholderia
Achromobacter
Delftia
Serratia
Haemophilus
Escherichia/Shigella
Rhodococcus
Ralstonia
Vibrio
Enhydrobacter
Novosphingobium
Bacteroides
Paracoccus
Micrococcus
Bradyrhizobium
Acinetobacter
Methylobacterium
Halomonas
Corynebacterium
Lactobacillus
Aeromonas
Acidovorax
Cloacibacterium
FC2a
Comp1
Comp2

Supplementary Figure S4: Heat map of ranked median abundances of each microbial genera present in the considered studies, showing geography-based clustering.

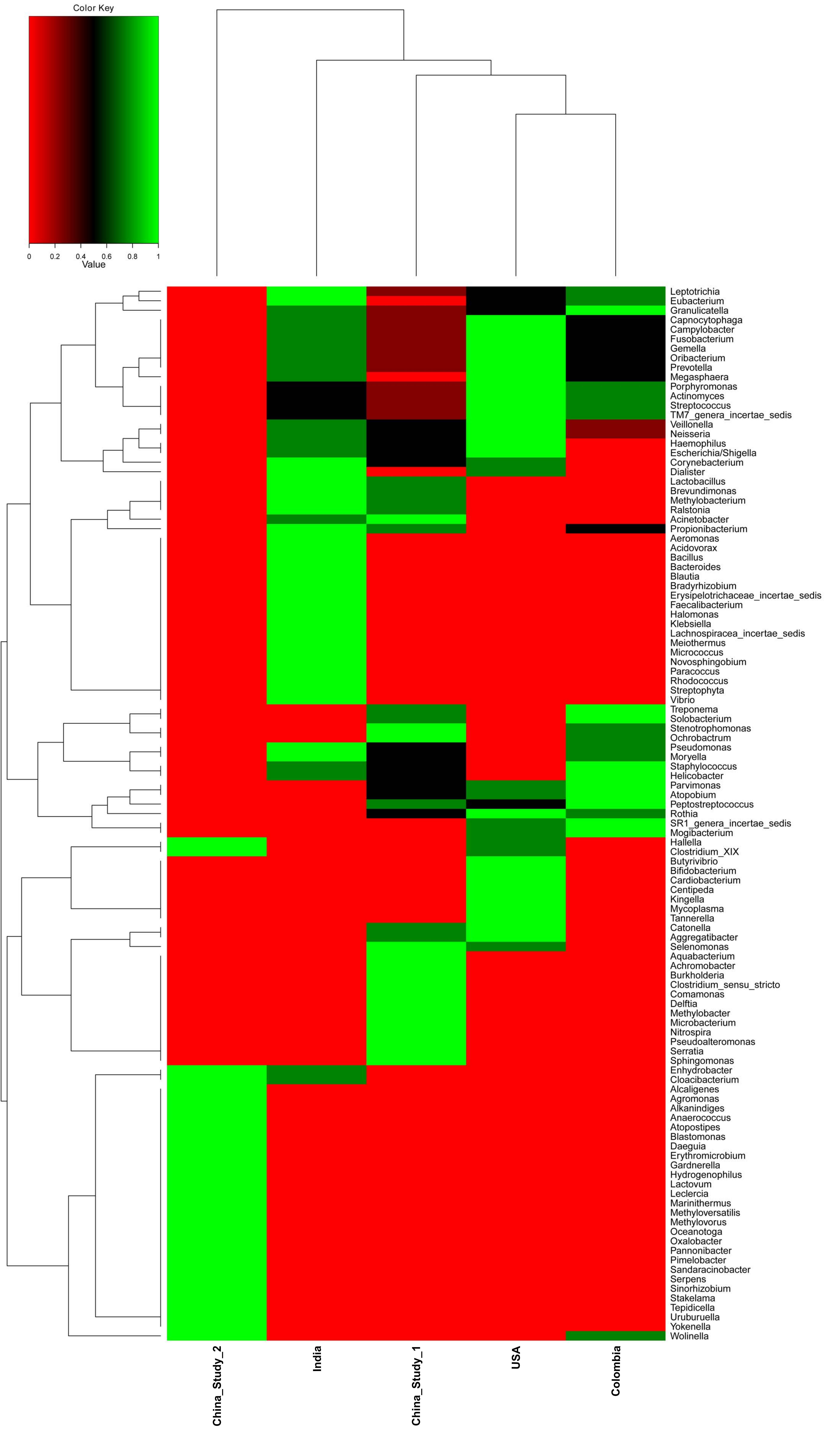

Color Key
0 0.2 0.4 0.6 0.8 1
Value
Leptotrichia
Eubacterium
Granulicatella
Capnocytophaga
Campylobacter
Fusobacterium
Gemella
Oribacterium
Prevotella
Megasphaera
Porphyromonas
Actinomyces
Streptococcus
TM7_genera_incertae_sedis
Veillonella
Neisseria
Haemophilus
Escherichia/Shigella
Corynebacterium
Dialister
Lactobacillus
Brevundimonas
Methylobacterium
Ralstonia
Acinetobacter
Propionibacterium
Aeromonas
Acidovorax
Bacillus
Bacteroides
Blautia
Bradyrhizobium
Erysipelotrichaceae_incertae_sedis
Faecalibacterium
Halomonas
Klebsiella
Lachnospiracea_incertae_sedis
Meiothermus
Micrococcus
Novosphingobium
Paracoccus
Rhodococcus
Streptophyta
Vibrio
Treponema
Solobacterium
Stenotrophomonas
Ochrobactrum
Pseudomonas
Moryella
Staphylococcus
Helicobacter
Parvimonas
Atopobium
Peptostreptococcus
Rothia
SR1_genera_incertae_sedis
Mogibacterium
Hallella
Clostridium_XIX
Butyrivibrio
Bifidobacterium
Cardiobacterium
Centipeda
Kingella
Mycoplasma
Tannerella
Catonella
Aggregatibacter
Selenomonas
Aquabacterium
Achromobacter
Burkholderia
Clostridium_sensu_stricto
Comamonas
Delftia
Methylobacter
Microbacterium
Nitrospira
Pseudoalteromonas
Serratia
Sphingomonas
Enhydrobacter
Cloacibacterium
Alcaligenes
Agromonas
Alkanindiges
Anaerococcus
Atopostipes
Blastomonas
Daeguia
Erythromicrobium
Gardnerella
Hydrogenophilus
Lactovum
Leclercia
Marinithermus
Methyloversatilis
Methylovorus
Oceanotoga
Oxalobacter
Pannonibacter
Pimelobacter
Sandaracinobacter
Serpens
Sinorhizobium
Stakelama
Tepidicella
Uruburuella
Yokenella
Wolinella
China_Study_2
India
China_Study_1
USA
Colombia

Supplementary Figure S5: Co-occurrence network of microbial genera present in samples from USA

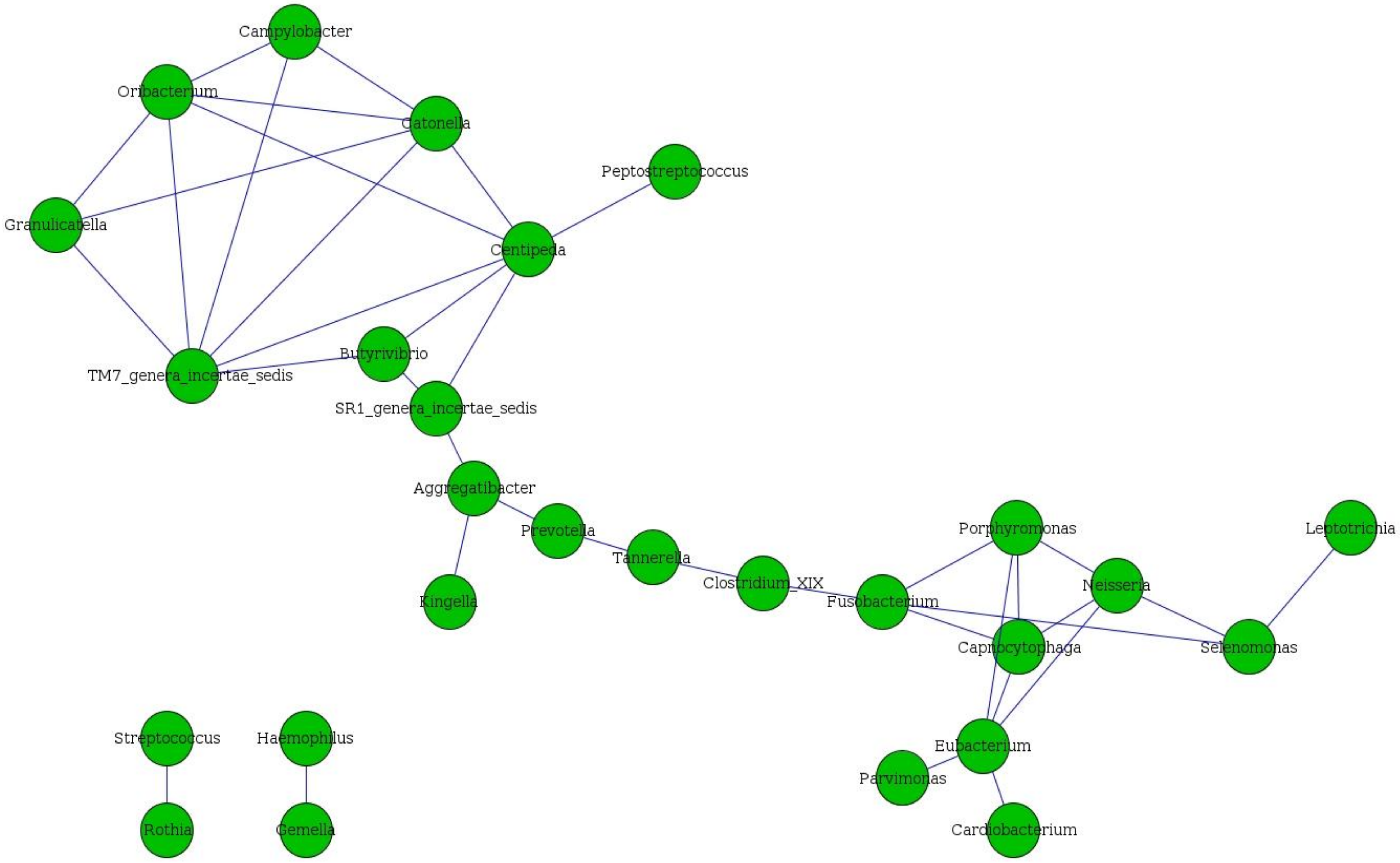

Supplementary Figure S6: Co-occurrence network of microbial genera present in samples from Chinese study 1

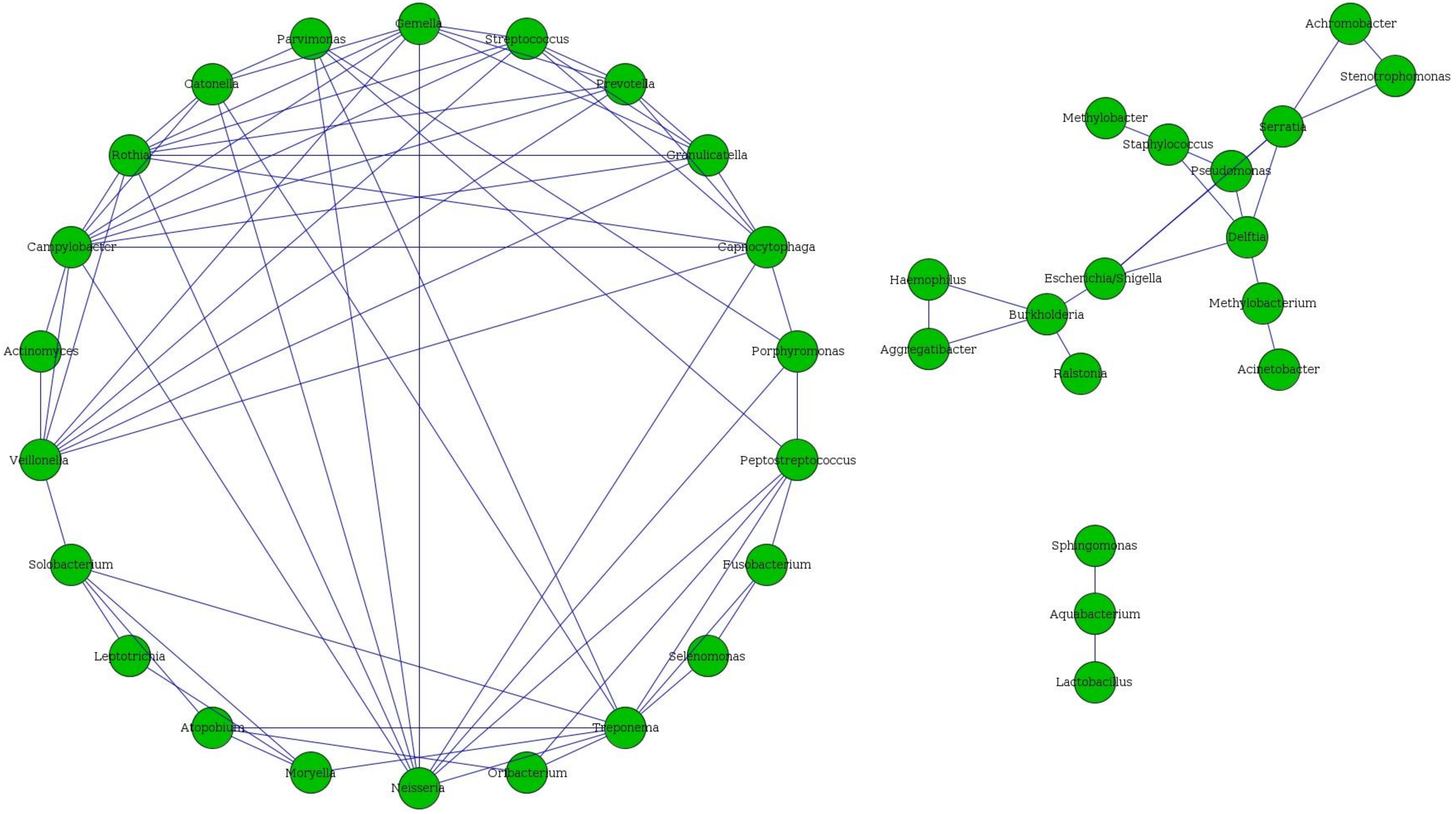

Supplementary Figure S7: Co-occurrence network of microbial genera present in samples from Chinese study 2

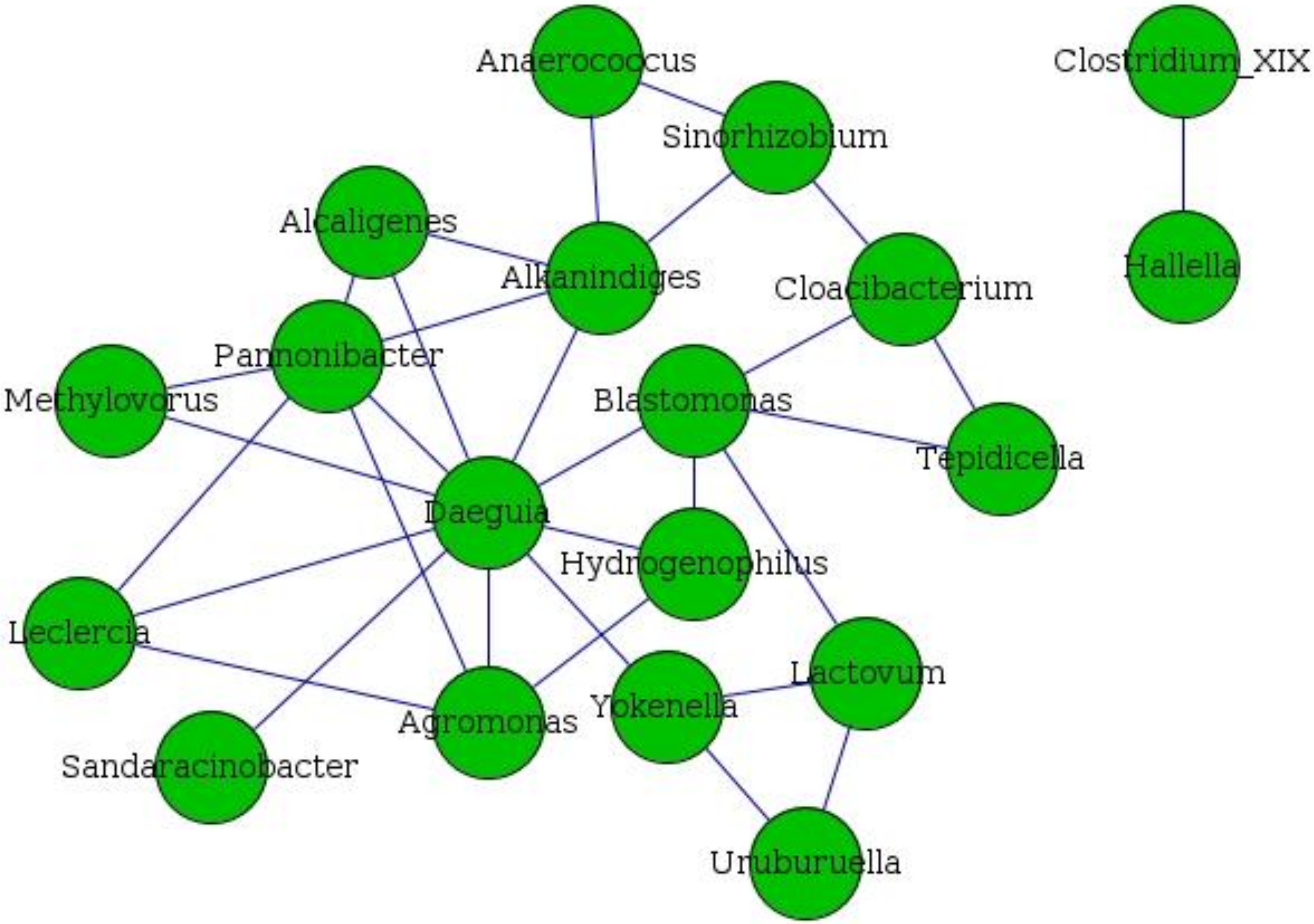

Supplementary Figure S8: Co-occurrence network of microbial genera present in samples from Colombian study

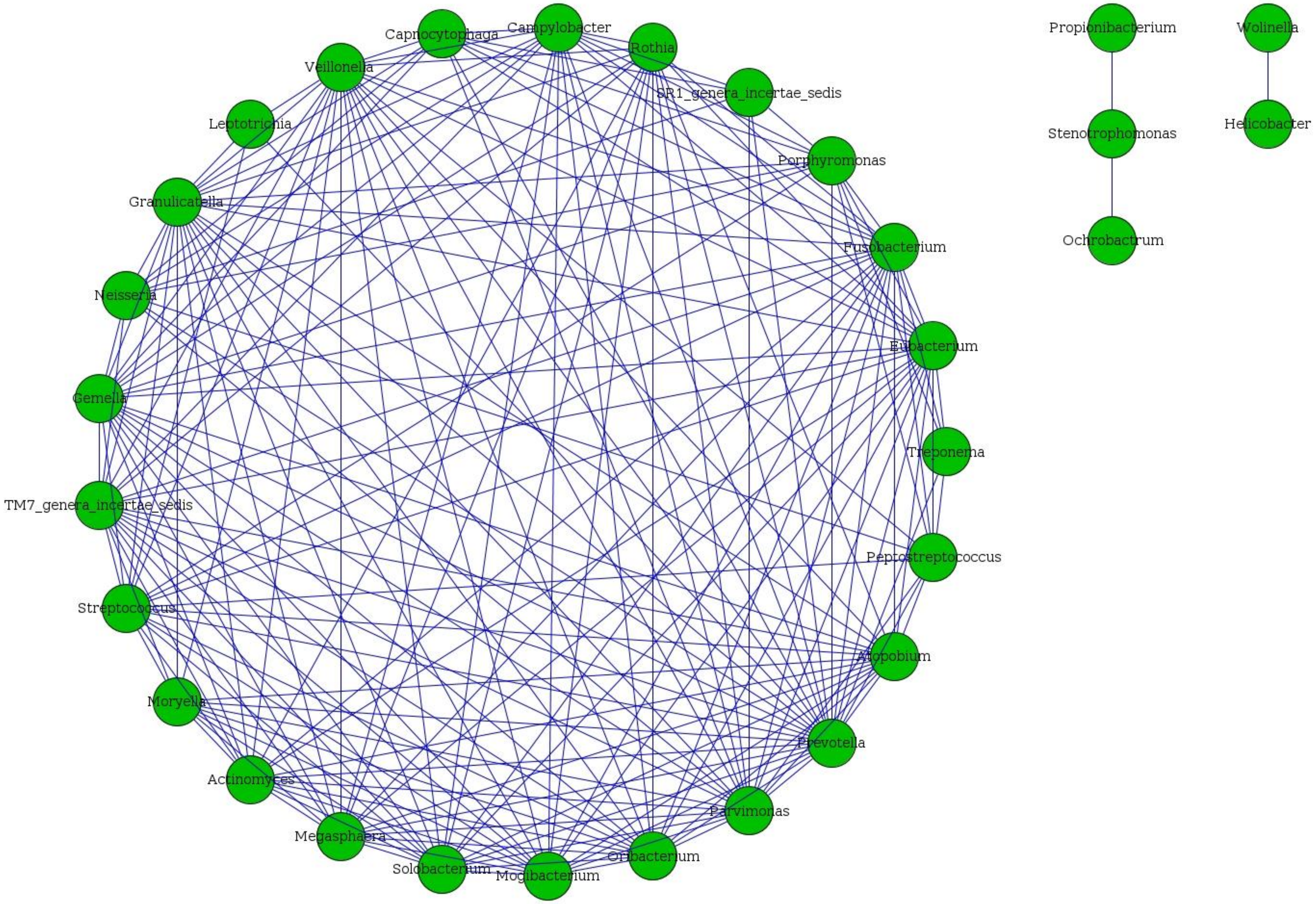

Supplementary Figure S9: Negative interaction network of microbial genera present in samples from USA

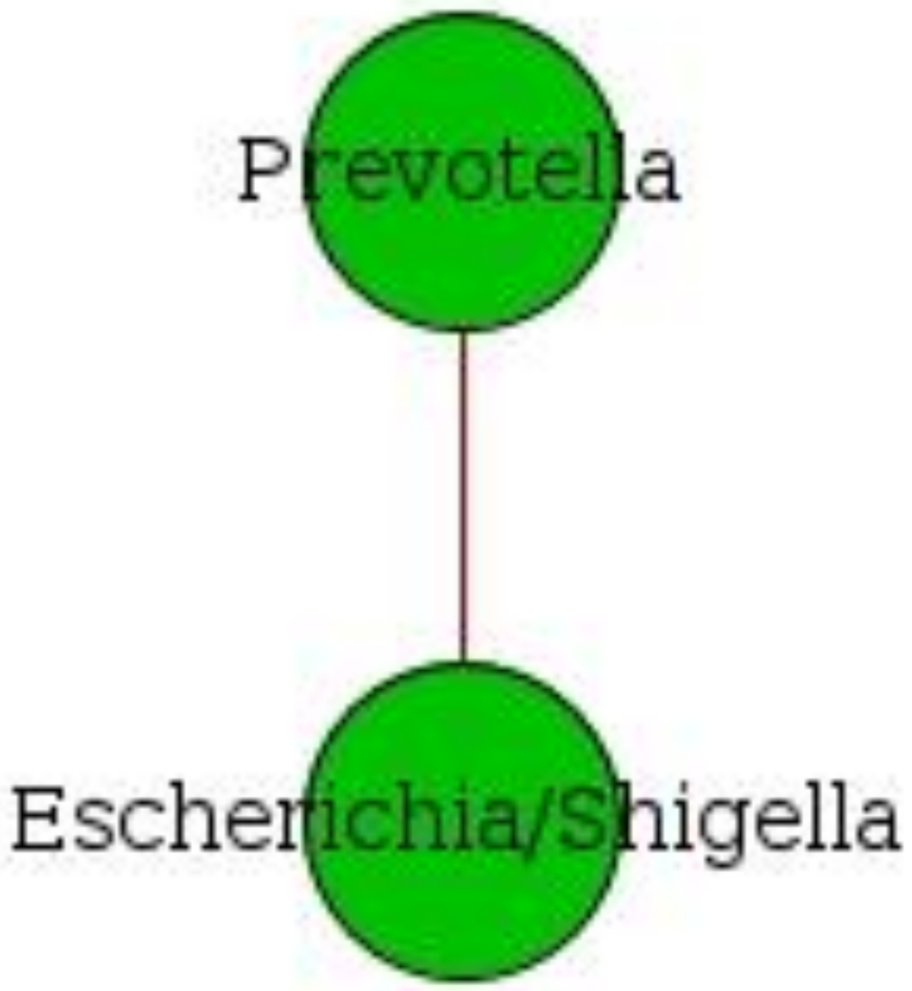

Supplementary Figure S10: Negative interaction network of microbial genera present in samples from Chinese study 1

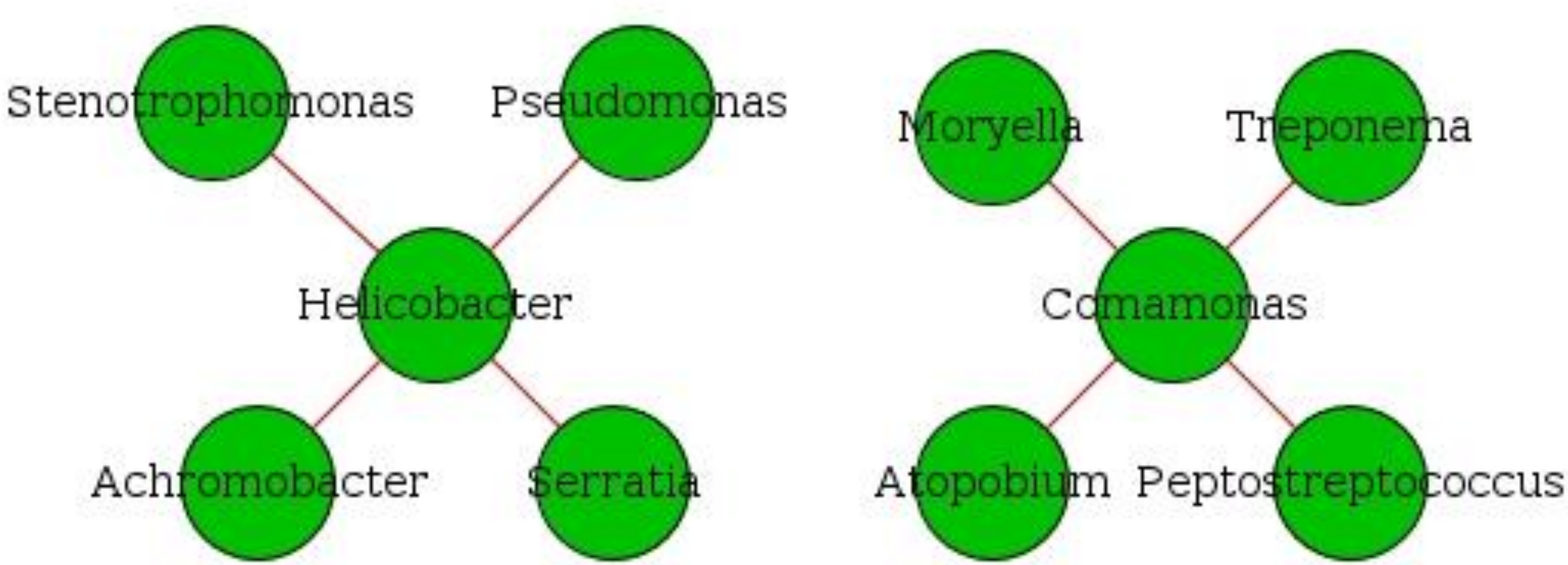

Supplementary Figure S11: Negative interaction network of microbial genera present in samples from Chinese study 2

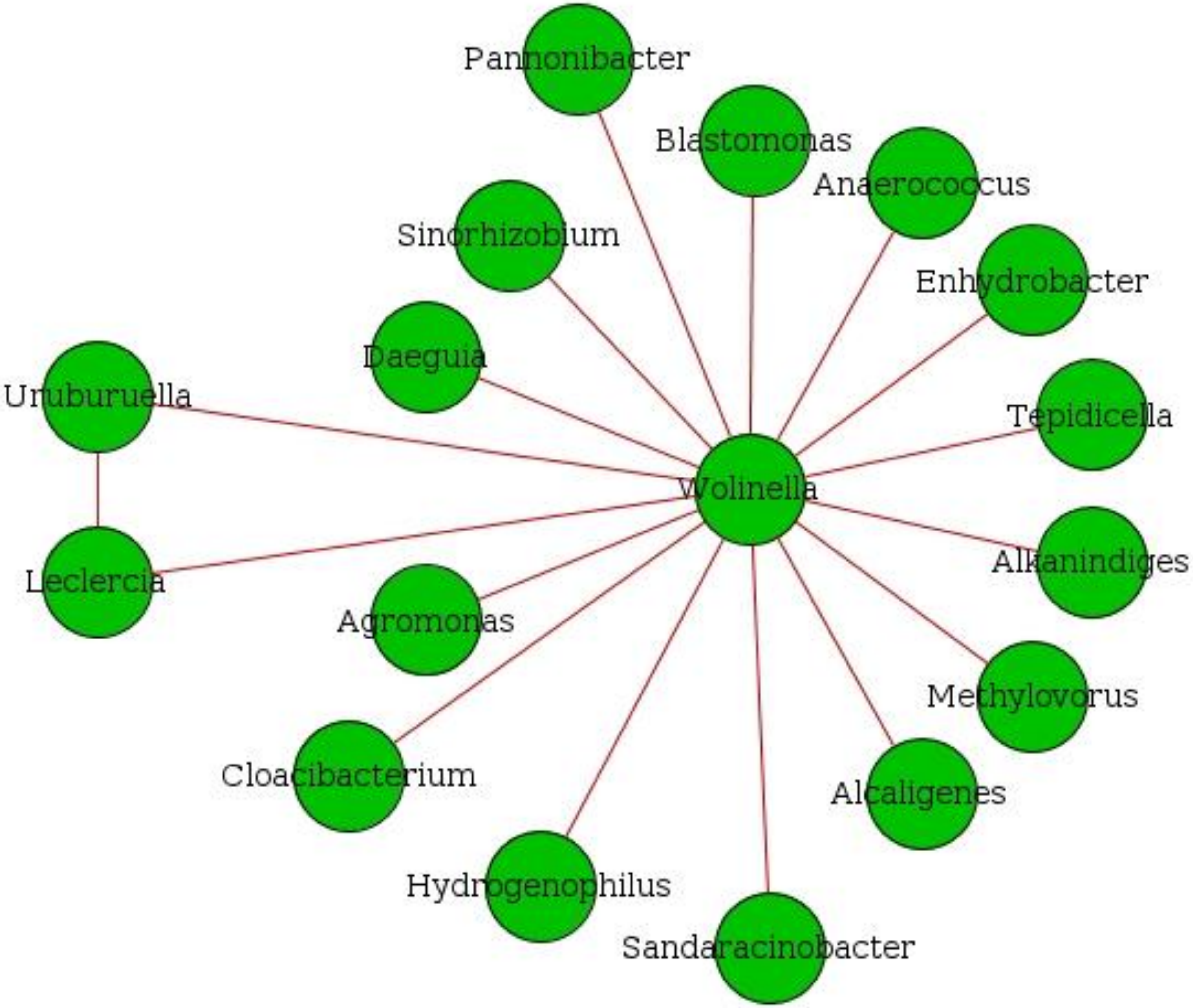

Supplementary Figure S12: Negative interaction network of microbial genera present in samples from Colombian study

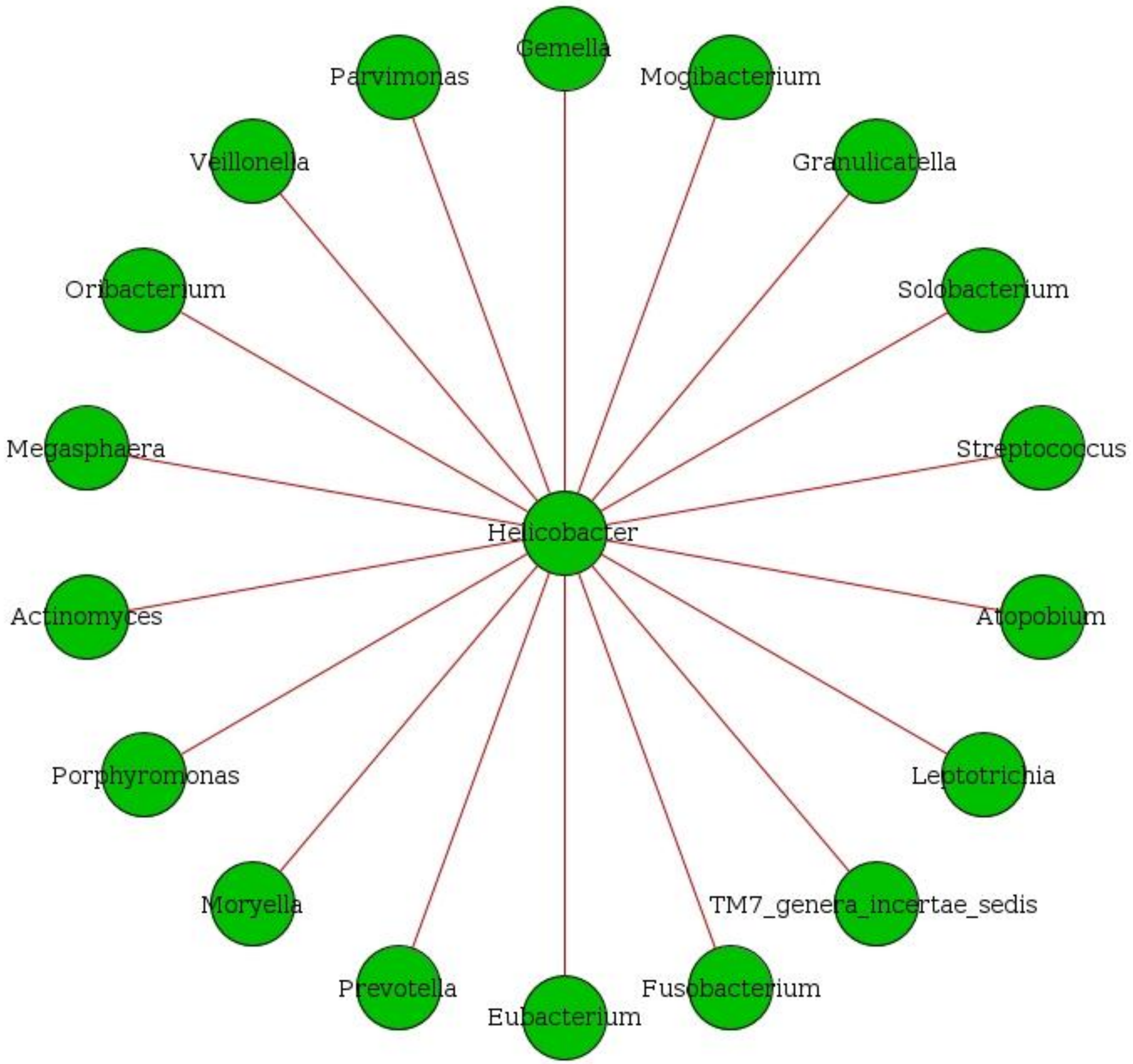

Supplementary Figure S13: Variation of Shannon diversity with Wolinella abundance, as calculated for Chinese study 2

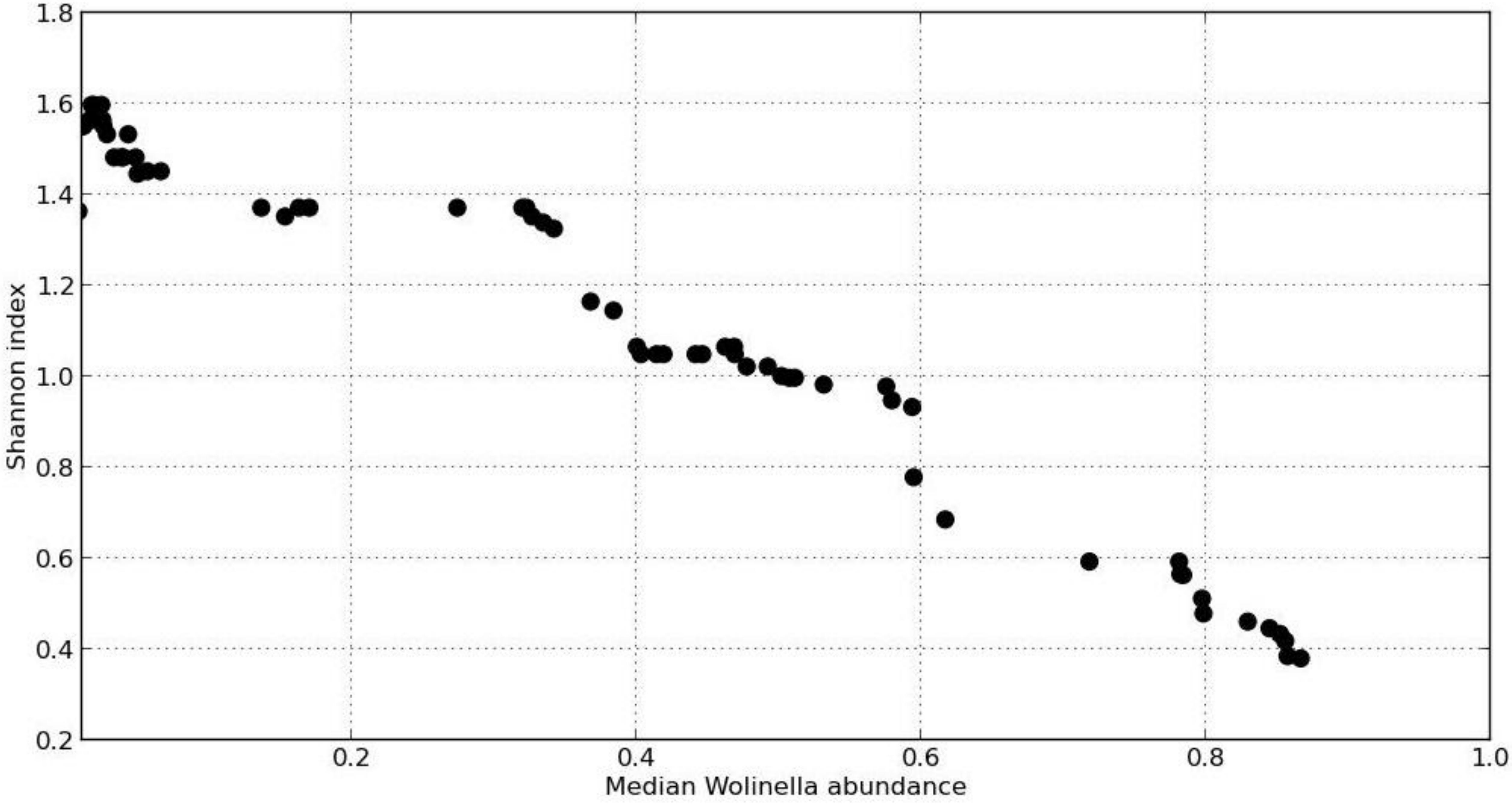

Supplementary Figure S14: Variation of Shannon diversity with Helicobacter abundance, as calculated for Colombian study

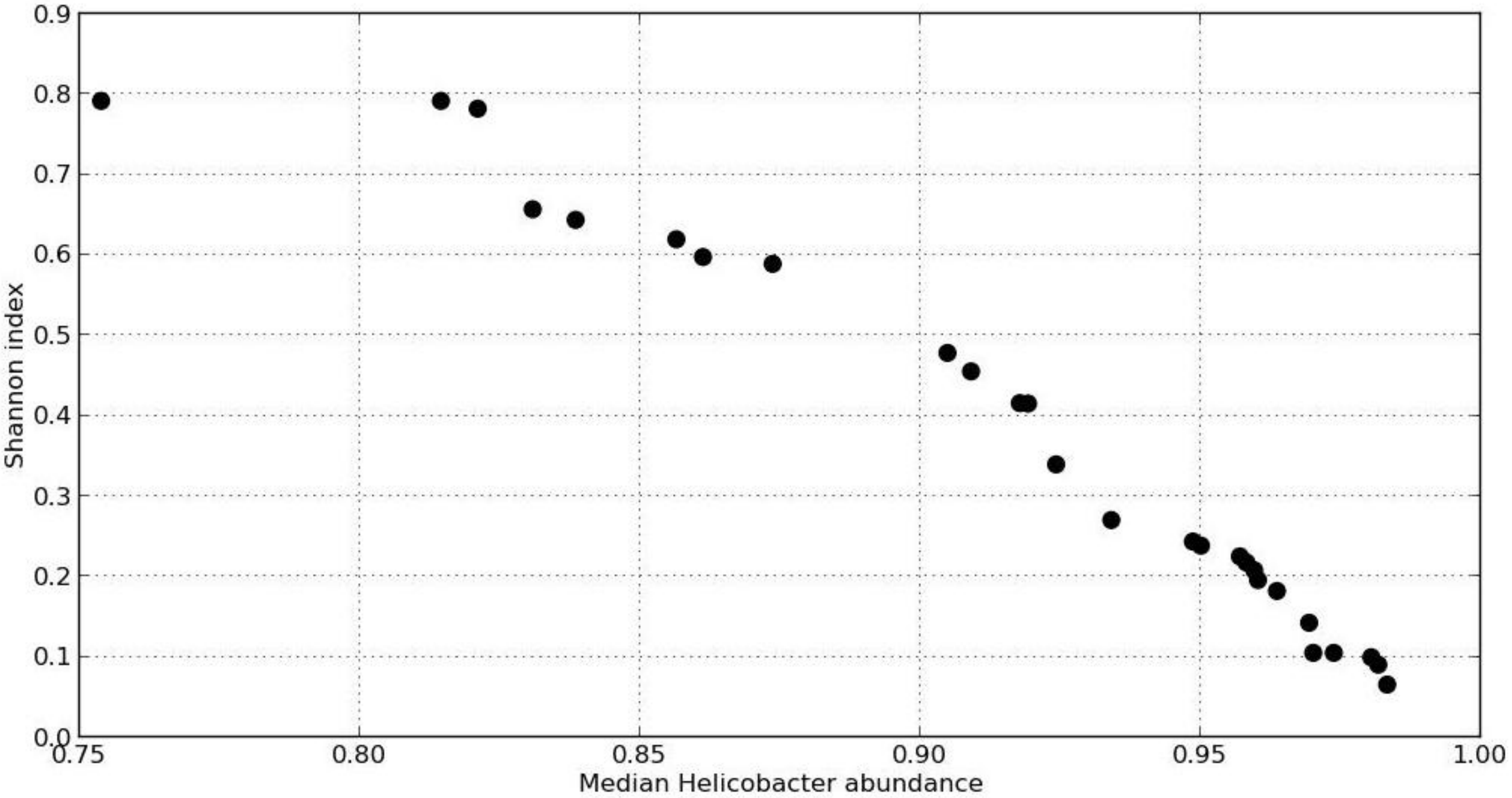

Supplementary Table S1: Sample IDs of subjects of reported Indian cohort, with corresponding age and gender information along with group affiliation.

SAMPLE ID	Age	Gender	Sample_Group	SAMPLE ID	Age	Gender	Sample_Group
S1	24	Male	G3	S36	53	Male	G4
S2	54	Male	G2	S37	22	Male	G4
S3	40	Male	G3	S38	56	Male	G4
S5	43	Female	G3	S41	45	Female	G4
S6	85	Female	G2	S42	45	Female	G4
S7	27	Male	G2	S43	55	Male	G4
S8	56	Male	G2	S44	25	Female	G1
S9	24	Male	G2	S45	34	Female	G1
S10	34	Male	G3	S46	45	Female	G1
S11	79	Male	G2	S47	45	Male	G1
S12	30	Female	G3	S48	41	Male	G4
S15	85	Male	G3	S49	60	Female	G4
S17	31	Female	G3	S50	28	Female	G1
S27	21	Male	G1	S51	45	Female	G1
S29	48	Male	G1	S52	48	Male	G4
S30	52	Male	G1	S53	43	Male	G4
S31	29	Male	G1	S54	38	Female	G4
S32	78	Male	G1	S55	52	Female	G3
S33	60	Female	G1	S56	46	Female	G4
S34	62	Male	G3				

Supplementary Table S2: Taxonomic lineage from classification of Operational Taxonomic Units (OTUs).

OTU_LIST	LINEAGE
OTU1	Bacteria;Proteobacteria;Betaproteobacteria;Burkholderiales;Comamonadaceae;Variovorax;uncultured Acidovorax sp.
OTU3	Bacteria;Bacteroidetes;Flavobacteria;Flavobacteriales;Flavobacteriaceae;Cloacibacterium;Flavobacteriaceae bacterium RB5-M2
OTU5	Bacteria;Bacteroidetes;Bacteroidia;Bacteroidales;Prevotellaceae;Prevotella;uncultured bacterium
OTU6	Bacteria;Proteobacteria;Betaproteobacteria;Neisseriales;Neisseriaceae;Neisseria;uncultured bacterium
OTU7	Bacteria;Actinobacteria;Actinobacteria;Micrococcales;Micrococcaceae;Micrococcus;Micrococcus sp. JN12
OTU8	Bacteria;Proteobacteria;Gammaproteobacteria;Oceanospirillales;Halomonadaceae;Halomonas;Halomonas sp. LAR6R1
OTU12	Bacteria;Firmicutes;Bacilli;Lactobacillales;Streptococcaceae;Streptococcus;uncultured Streptococcus sp.
OTU14	Bacteria;Firmicutes;Negativicutes;Selenomonadales;Veillonellaceae;Veillonella;Veillonella sp. S4-C10
OTU15	Bacteria;Firmicutes;Clostridia;Clostridiales;Ruminococcaceae;Faecalibacterium;uncultured bacterium
OTU16	Bacteria;Proteobacteria;Gammaproteobacteria;Enterobacteriales;Enterobacteriaceae;Escherichia-Shigella;Escherichia coli
OTU17	Bacteria;Proteobacteria;Gammaproteobacteria;Pasteurellales;Pasteurellaceae;Actinobacillus;uncultured bacterium
OTU20	Bacteria;Fusobacteria;Fusobacteriia;Fusobacteriales;Fusobacteriaceae;Fusobacterium;uncultured bacterium
OTU23	Bacteria;Firmicutes;Bacilli;Lactobacillales;Carnobacteriaceae;Carnobacterium;uncultured bacterium
OTU27	Bacteria;Proteobacteria;Alphaproteobacteria;Rhizobiales;Bradyrhizobiaceae;Bradyrhizobium;uncultured Bradyrhizobium sp.
OTU31	Bacteria;Proteobacteria;Epsilonproteobacteria;Campylobacterales;Helicobacteraceae;Helicobacter;Helicobacter pylori
OTU33	Bacteria;Candidate division TM7;uncultured bacterium
OTU37	Bacteria;Bacteroidetes;Bacteroidia;Bacteroidales;Bacteroidaceae;Bacteroides;bacterium NLAE-zl-G257
OTU44	Bacteria;Proteobacteria;Alphaproteobacteria;Rhodobacterales;Rhodobacteraceae;Paracoccus;Paracoccus sp. CS4
OTU56	Bacteria;SM2F11;uncultured organism
OTU60	Bacteria;Deinococcus-Thermus;Deinococci;Thermales;Thermaceae;Meiothermus;Meiothermus silvanus DSM 9946
OTU61	Bacteria;Actinobacteria;Actinobacteria;Streptomycetales;Streptomycetaceae;Streptomyces;Streptomyces globosus
OTU73	Bacteria;Bacteroidetes;Bacteroidia;Bacteroidales;Prevotellaceae;Prevotella;uncultured bacterium
OTU76	Bacteria;Cyanobacteria;Chloroplast;Tofieldia calyculata
OTU86	Bacteria;Proteobacteria;Alphaproteobacteria;Sphingomonadales;Sphingomonadaceae;Sphingopyxis;uncultured bacterium
OTU100	Bacteria;Proteobacteria;Gammaproteobacteria;Chromatiales;Chromatiaceae;Nitrosococcus;uncultured bacterium
OTU111	Bacteria;Actinobacteria;Actinobacteria;Streptomycetales;Streptomycetaceae;Streptomyces;Streptomyces globosus
OTU133	Bacteria;Firmicutes;Clostridia;Clostridiales;Ruminococcaceae;uncultured;uncultured rumen bacterium
OTU186	Bacteria;Proteobacteria;Gammaproteobacteria;Pseudomonadales;Pseudomonadaceae;Pseudomonas;uncultured bacterium
OTU264	Bacteria;Bacteroidetes;Bacteroidia;Bacteroidales;Porphyromonadaceae;Porphyromonas;uncultured bacterium
OTU345	Bacteria;Bacteroidetes;Bacteroidia;Bacteroidales;Prevotellaceae;Prevotella;Prevotella sp. ICM55
OTU350	Bacteria;Firmicutes;Clostridia;Clostridiales;Family XIII Incertae Sedis;Incertae Sedis;[Eubacterium] sulci
OTU357	Bacteria;Bacteroidetes;Bacteroidia;Bacteroidales;Porphyromonadaceae;Porphyromonas;Porphyromonas endodontalis
OTU358	Bacteria;Proteobacteria;Alphaproteobacteria;Rhizobiales;Methylobacteriaceae;Methylobacterium;Methylobacteriaceae bacterium KVD-1982-01
OTU359	Bacteria;Bacteroidetes;Bacteroidia;Bacteroidales;Prevotellaceae;Prevotella;uncultured bacterium
OTU363	Bacteria;Actinobacteria;Actinobacteria;Propionibacteriales;Propionibacteriaceae;Propionibacterium;uncultured bacterium
OTU372	Bacteria;Proteobacteria;Gammaproteobacteria;Pseudomonadales;Moraxellaceae;Acinetobacter;Acinetobacter sp.
OTU373	Bacteria;Proteobacteria;Gammaproteobacteria;Pseudomonadales;Moraxellaceae;Enhydrobacter;uncultured bacterium
OTU375	Bacteria;Bacteroidetes;Bacteroidia;Bacteroidales;Prevotellaceae;Prevotella;Prevotella nanceiensis
OTU381	Bacteria;Bacteroidetes;Bacteroidia;Bacteroidales;Prevotellaceae;Prevotella;uncultured Bacteroidetes bacterium
OTU385	Bacteria;Proteobacteria;Epsilonproteobacteria;Campylobacterales;Campylobacteraceae;Campylobacter;Campylobacter concisus UNSWCD
OTU386	Bacteria;Firmicutes;Bacilli;Bacillales;Family XI Incertae Sedis;Gemella;Gemella sp. 933-88
OTU394	Bacteria;Proteobacteria;Alphaproteobacteria;Sphingomonadales;Sphingomonadaceae;Novosphingobium;Sphingomonas sp. SL21
OTU400	Bacteria;Proteobacteria;Alphaproteobacteria;Caulobacterales;Caulobacteraceae;Brevundimonas;uncultured Brevundimonas sp.

OTU404	Bacteria;Firmicutes;Negativicutes;Selenomonadales;Veillonellaceae;Dialister;uncultured organism
OTU455	Bacteria;Proteobacteria;Betaproteobacteria;Burkholderiales;Burkholderiaceae;Ralstonia;unidentified marine bacterioplankton
OTU456	Bacteria;Actinobacteria;Actinobacteria;Actinomycetales;Actinomycetaceae;Actinomyces;uncultured bacterium
OTU463	Bacteria;Bacteroidetes;Bacteroidia;Bacteroidales;Prevotellaceae;Prevotella;uncultured bacterium
OTU468	Bacteria;Firmicutes;Bacilli;Lactobacillales;Lactobacillaceae;Lactobacillus;uncultured bacterium
OTU481	Bacteria;Actinobacteria;Actinobacteria;Corynebacteriales;Corynebacteriaceae;Corynebacterium;uncultured bacterium
OTU482	Bacteria;Actinobacteria;Actinobacteria;Corynebacteriales;Nocardiaceae;Rhodococcus;Rhodococcus erythropolis
OTU520	Bacteria;Firmicutes;Bacilli;Lactobacillales;Lactobacillaceae;Lactobacillus;uncultured bacterium
OTU552	Bacteria;Firmicutes;Clostridia;Clostridiales;Lachnospiraceae;Stomatobaculum;Stomatobaculum longum
OTU553	Bacteria;Proteobacteria;Gammaproteobacteria;Vibrionales;Vibrionaceae;Vibrio;Vibrio cholerae CP1048(21)
OTU560	Bacteria;Bacteroidetes;Bacteroidia;Bacteroidales;Prevotellaceae;Prevotella;uncultured bacterium
OTU564	Bacteria;Firmicutes;Negativicutes;Selenomonadales;Veillonellaceae;Megasphaera;Megasphaera micronuciformis F0359
OTU565	Bacteria;Actinobacteria;Actinobacteria;Corynebacteriales;Corynebacteriaceae;Corynebacterium;uncultured bacterium
OTU579	Bacteria;Firmicutes;Clostridia;Clostridiales;Lachnospiraceae;Oribacterium;uncultured bacterium
OTU585	Bacteria;Firmicutes;Clostridia;Clostridiales;Lachnospiraceae;Lachnoanaerobaculum;uncultured organism
OTU611	Bacteria;Proteobacteria;Gammaproteobacteria;Aeromonadales;Aeromonadaceae;Aeromonas;Aeromonas aquariorum
OTU613	Bacteria;Firmicutes;Bacilli;Bacillales;Staphylococcaceae;Staphylococcus;Staphylococcus caprae
OTU659	Bacteria;Bacteroidetes;Bacteroidia;Bacteroidales;Prevotellaceae;Prevotella;uncultured bacterium
OTU661	Bacteria;Bacteroidetes;Bacteroidia;Bacteroidales;Prevotellaceae;Prevotella;uncultured bacterium
OTU668	Bacteria;Firmicutes;Bacilli;Lactobacillales;Lactobacillaceae;Lactobacillus;uncultured bacterium
OTU679	Bacteria;Proteobacteria;Gammaproteobacteria;Pseudomonadales;Pseudomonadaceae;Pseudomonas;uncultured Pseudomonas sp.
OTU704	Bacteria;Bacteroidetes;Bacteroidia;Bacteroidales;Prevotellaceae;Prevotella;Prevotella nigrescens ATCC 33563
OTU729	Bacteria;Firmicutes;Bacilli;Lactobacillales;Streptococcaceae;Streptococcus;Streptococcus salivarius
OTU731	Bacteria;Proteobacteria;Gammaproteobacteria;Pseudomonadales;Pseudomonadaceae;Pseudomonas;Pseudomonas stutzeri
OTU732	Bacteria;Proteobacteria;Gammaproteobacteria;Pasteurellales;Pasteurellaceae;Haemophilus;uncultured bacterium
OTU757	Bacteria;Actinobacteria;Actinobacteria;Micrococcales;Micrococcaceae;Arthrobacter;Kocuria sp. SS14.15
OTU763	Bacteria;Proteobacteria;Alphaproteobacteria;Sphingomonadales;Sphingomonadaceae;Novosphingobium;uncultured bacterium

Supplementary Table S3: Microbial genera and corresponding lowest taxa affiliation identified at OTU level.

Genera	Lowest Taxa Level
Acidovorax	Acidovorax_sp
Acinetobacter	Acinetobacter_sp
Actinomyces	Actinomyces_uncultured_bacterium
Aeromonas	Aeromonas_aquariorum
Bacteroides	Bacteroides_bacterium_NLAE-zl-G257
Bradyrhizobium	uncultured_Bradyrhizobium_sp
Brevundimonas	uncultured_Brevundimonas_sp
Campylobacter	Campylobacter_concisus_UNSWCD
Corynebacterium	Corynebacterium_uncultured_bacterium1
Corynebacterium	Corynebacterium_uncultured_bacterium2
Dialister	Dialister_uncultured_organism
Enhydrobacter	Enhydrobacter_uncultured_bacterium
Escherichia	Escherichia_coli
Faecalibacterium	Faecalibacterium_bacterium
Fusobacterium	Fusobacterium_uncultured_bacterium
Gemella	Gemella_sp_933-88
Haemophilus	Haemophilus_uncultured_bacterium
Halomonas	Halomonas_sp_LAR6R1
Helicobacter	Helicobacter_pylori
Lachnospiracea_incertae_sedis	Lachnoanaerobaculum_uncultured_organism
Lactobacillus	Lactobacillus_uncultured_bacterium1
Lactobacillus	Lactobacillus_uncultured_bacterium2
Lactobacillus	Lactobacillus_uncultured_bacterium3
Megasphaera	Megasphaera_micronuciformis_F0359
Meiothermus	Meiothermus_silvanus_DSM_9946
Micrococcus	Micrococcus_sp_JN12
Neisseria	Neisseria_uncultured_bacterium
Novosphingobium	Novosphingobium_uncultured_bacterium
Oribacterium	Oribacterium_uncultured_bacterium
Paracoccus	Paracoccus_sp_CS4
Porphyromonas	Porphyromonas_uncultured_bacterium
Prevotella	Prevotella_uncultured_bacterium1
Prevotella	Prevotella_uncultured_bacterium2
Prevotella	Prevotella_sp_ICM55
Prevotella	Prevotella_uncultured_bacterium3
Prevotella	Prevotella_nanceiensis
Prevotella	Prevotella_uncultured_bacterium4
Prevotella	Prevotella_uncultured_bacterium5
Prevotella	Prevotella_uncultured_bacterium6
Prevotella	Prevotella_uncultured_bacterium7
Prevotella	Prevotella_uncultured_bacterium8
Prevotella	Prevotella_nigrescens_ATCC_33563

Propionibacterium	Propionibacterium_uncultured_bacterium
Pseudomonas	Pseudomonas_uncultured_bacterium
Pseudomonas	uncultured_Pseudomonas_sp
Pseudomonas	Pseudomonas_stutzeri
Ralstonia	Ralstonia_unidentified_marine_bacterioplankton
Rhodococcus	Rhodococcus_erythropolis
Staphylococcus	Staphylococcus_caprae
Streptococcus	uncultured_Streptococcus_sp
Streptococcus	Streptococcus_salivarius
TM7_genera_incertae_sedis	TM7_uncultured_bacterium
Veillonella	Veillonella_sp_S4-C10
Vibrio	Vibrio_cholerae_CP1048(21)

Supplementary Table S4: Microbial genera identified in reported Indian cohort and corresponding cluster affiliations.

Genus	Cluster	Genus	Cluster
Actinomyces	C1	Acinetobacter	C2
Bacillus	C1	Aeromonas	C2
Campylobacter	C1	Bacteroides	C2
Capnocytophaga	C1	Blautia	C2
Dialister	C1	Bradyrhizobium	C2
Escherichia/Shigella	C1	Brevundimonas	C2
Eubacterium	C1	Cloacibacterium	C2
Fusobacterium	C1	Corynebacterium	C2
Gemella	C1	Enhydrobacter	C2
Granulicatella	C1	Erysipelotrichaceae_incertae_sedis	C2
Haemophilus	C1	Faecalibacterium	C2
Leptotrichia	C1	Halomonas	C2
Megasphaera	C1	Helicobacter	C2
Moryella	C1	Klebsiella	C2
Neisseria	C1	Lachnospiracea_incertae_sedis	C2
Novosphingobium	C1	Lactobacillus	C2
Oribacterium	C1	Meiothermus	C2
Porphyromonas	C1	Methylobacterium	C2
Prevotella	C1	Micrococcus	C2
Ralstonia	C1	Paracoccus	C2
Streptococcus	C1	Propionibacterium	C2
TM7_genera_incertae_sedis	C1	Pseudomonas	C2
Veillonella	C1	Rhodococcus	C2
Vibrio	C1	Staphylococcus	C2
Acidovorax	C2	Streptophyta	C2

Supplementary Table S5: Sample ids and sequencing information of each sample.

Samples	Number of Sequences	Mean_Length	Std. Dev. (Length)	PRIMER NO	BARCODE NAME	BARCODE SEQUENCE
S1	28172	865.63	57.42	265	265(926R34)	ACGGCTC
S2	21460	851.68	101.35	266	266(926R35)	TGCGTTC
S3	21464	846.48	69.38	267	267(926R36)	TCTCGAC
S5	12018	869.27	54.3	269	269(926R38)	ACTCCTC
S6	2235	832.65	117.07	270	270(926R39)	TTCCTGC
S7	20859	869.98	71.85	271	271(926R40)	TTCATAC
S8	21600	850.28	96.18	272	272(926R41)	CGTCGTC
S9	5734	854.26	84.2	273	273(926R42)	AAGGCAC
S10	25124	870.42	58.56	274	274(926R43)	AACAACTC
S11	7847	855.38	102.08	275	275(926R44)	ACACGGAC
S12	11422	870.59	59.63	276	276(926R45)	TGCCGAAC
S15	10791	867.33	61.32	279	279(926R48)	CCGGCCAC
S17	93926	866.87	56.88	281	281(926R50)	TCTCCGTC
S27	26981	844.32	102.22	233	233(926R2)	CGCAAC
S29	35996	853.61	94.41	235	235(926R4)	ACTTGC
S30	76213	847.69	99.33	236	236(926R5)	TCACAC
S31	23573	851.89	92.07	237	237(926R6)	CGTGAC
S32	23539	848.65	97.76	238	238(926R7)	ACGCGC
S33	38886	852.66	97.6	239	239(926R8)	CCTCTC
S34	15962	842.32	91.37	240	240(926R9)	ACTCAC
S36	18005	843.74	93.09	242	242(926R11)	CGACTC
S37	17017	848.13	88.33	243	243(926R12)	AGCTTC
S38	17024	831.12	108.76	244	244(926R13)	AAGCCG
S41	19251	850.01	86.28	247	247(926R16)	TATCAAC
S42	14717	833.13	106.95	248	248(926R17)	AGGCGGC
S43	15626	846.68	97.37	249	249(926R18)	CGGTATC
S44	16486	822.07	115.49	250	250(926R19)	TGACGAC
S45	19710	844.75	94.84	251	251(926R20)	ACAAGGC
S46	14008	835.19	103.69	252	252(926R21)	AGACCTC
S47	14555	835.08	110.58	253	253(926R22)	ATACCAC
S48	26128	840.44	96.42	254	254(926R23)	TCGCGGC
S49	23249	860.51	69.57	255	255(926R24)	ATCTTAC
S50	14498	847.4	97.1	256	256(926R25)	AACCAGC
S51	12397	845.22	95.99	257	257(926R26)	TTCGAGC
S52	13412	848.69	87.77	258	258(926R27)	AAGGTGC
S53	18172	836.62	100.89	259	259(926R28)	TCTTGGC
S54	15888	826.09	99.15	260	260(926R29)	TAATCTC
S55	20218	850.24	83.09	261	261(926R30)	TCACCTC
S56	60072	849.32	86.14	262	262(926R31)	TCCGCTC
**Total Sequences**	941415					
**Filtered Sequences**	940591					
**Sequences fetched by tags**	894235					
